# Lopinavir–ritonavir in patients admitted to hospital with COVID-19 (RECOVERY): a randomised, controlled, open-label, platform trial

**DOI:** 10.1016/S0140-6736(20)32013-4

**Published:** 2020-10-24

**Authors:** RECOVERY Collaborative Group, Peter W Horby, Marion Mafham, Jennifer L Bell, Louise Linsell, Natalie Staplin, Jonathan Emberson, Adrian Palfreeman, Jason Raw, Einas Elmahi, Benjamin Prudon, Christopher Green, Simon Carley, David Chadwick, Matthew Davies, Matthew P Wise, J Kenneth Baillie, Lucy C Chappell, Saul N Faust, Thomas Jaki, Katie Jefferey, Wei Shen Lim, Alan Montgomery, Kathryn Rowan, Edmund Juszczak, Richard Haynes, Martin J Landray

## Abstract

**Background:**

Lopinavir–ritonavir has been proposed as a treatment for COVID-19 on the basis of in vitro activity, preclinical studies, and observational studies. Here, we report the results of a randomised trial to assess whether lopinavir–ritonavir improves outcomes in patients admitted to hospital with COVID-19.

**Methods:**

In this randomised, controlled, open-label, platform trial, a range of possible treatments was compared with usual care in patients admitted to hospital with COVID-19. The trial is underway at 176 hospitals in the UK. Eligible and consenting patients were randomly allocated to either usual standard of care alone or usual standard of care plus lopinavir–ritonavir (400 mg and 100 mg, respectively) by mouth for 10 days or until discharge (or one of the other RECOVERY treatment groups: hydroxychloroquine, dexamethasone, or azithromycin) using web-based simple (unstratified) randomisation with allocation concealment. Randomisation to usual care was twice that of any of the active treatment groups (eg, 2:1 in favour of usual care if the patient was eligible for only one active group, 2:1:1 if the patient was eligible for two active groups). The primary outcome was 28-day all-cause mortality. Analyses were done on an intention-to-treat basis in all randomly assigned participants. The trial is registered with ISRCTN, 50189673, and ClinicalTrials.gov, NCT04381936.

**Findings:**

Between March 19, 2020, and June 29, 2020, 1616 patients were randomly allocated to receive lopinavir–ritonavir and 3424 patients to receive usual care. Overall, 374 (23%) patients allocated to lopinavir–ritonavir and 767 (22%) patients allocated to usual care died within 28 days (rate ratio 1·03, 95% CI 0·91–1·17; p=0·60). Results were consistent across all prespecified subgroups of patients. We observed no significant difference in time until discharge alive from hospital (median 11 days [IQR 5 to >28] in both groups) or the proportion of patients discharged from hospital alive within 28 days (rate ratio 0·98, 95% CI 0·91–1·05; p=0·53). Among patients not on invasive mechanical ventilation at baseline, there was no significant difference in the proportion who met the composite endpoint of invasive mechanical ventilation or death (risk ratio 1·09, 95% CI 0·99–1·20; p=0·092).

**Interpretation:**

In patients admitted to hospital with COVID-19, lopinavir–ritonavir was not associated with reductions in 28-day mortality, duration of hospital stay, or risk of progressing to invasive mechanical ventilation or death. These findings do not support the use of lopinavir–ritonavir for treatment of patients admitted to hospital with COVID-19.

**Funding:**

Medical Research Council and National Institute for Health Research.

## Introduction

Severe acute respiratory syndrome coronavirus 2 (SARS-CoV-2), the cause of COVID-19, emerged in China in late 2019 from a zoonotic source. Most COVID-19 infections are either asymptomatic or result in only mild disease.^1^ However, a proportion of infected individuals develop a respiratory illness that requires hospital care, which can progress to critical illness with hypoxaemic respiratory failure that requires prolonged ventilatory support. Among patients with COVID-19 admitted to UK hospitals, the case fatality rate is over 26%, and is in excess of 37% in patients who require invasive mechanical ventilation.^2^

The drug combination lopinavir–ritonavir has been suggested as an antiviral treatment for COVID-19.^3^ Lopinavir is a HIV-1 protease inhibitor, which is combined with ritonavir to increase its plasma half-life. Lopinavir is also an inhibitor of the severe acute respiratory syndrome coronavirus (SARS-CoV) main protease, which is critical for replication and appears to be highly conserved in SARS-CoV-2.^4^, ^5^ Lopinavir has in vitro inhibitory activity against SARS-CoV, SARS-CoV-2, and Middle East respiratory syndrome (MERS) coronavirus.^6^, ^7^, ^8^, ^9^ In a marmoset model of MERS, lopinavir–ritonavir improved clinical, radiological, and pathological outcomes and reduced viral loads.^10^ A study of lopinavir–ritonavir in a ferret model of COVID-19 found reduced clinical symptoms in treated animals but no effect on virus titres.^11^

In patients with severe acute respiratory syndrome, a historically controlled study suggested that addition of lopinavir–ritonavir to ribavirin reduced the risk of adverse clinical outcomes and viral load.^12^ Although some observational studies in patients with COVID-19 have reported that lopinavir–ritonavir is associated with a shorter duration of viral shedding^13^ and fever,^14^ other studies have reported no such effects.^15^, ^16^ A previous randomised trial of lopinavir–ritonavir among 199 patients admitted to hospital with COVID-19 showed no improvement in viral load, duration of hospital stay, or mortality.^17^ However, the trial was too small to rule out the possibility of clinically relevant benefits and commentators recommended larger randomised trials to confirm or refute the lack of effect.^18^ Here, we report the results of a randomised trial to assess whether lopinavir–ritonavir improves clinical outcomes in patients admitted to hospital with COVID-19.

**Research in context**

**Evidence before this study**

We searched PubMed from inception up to July 23, 2020, for clinical trials published in English evaluating the effect of lopinavir–ritonavir among patients with laboratory-confirmed COVID-19 using the search terms (“COVID-19”[All Fields] OR “2019-nCoV”[All Fields]) OR “SARS-CoV-2”[All Fields]) AND (“lopinavir”[All Fields] OR “ritonavir”[All Fields]) (filters: Clinical Trial, Randomized Controlled Trial). We identified only one randomised clinical trial that compared lopinavir–ritonavir with usual care in patients with COVID-19. This trial assigned 99 patients who had been admitted to hospital with COVID-19 to a lopinavir–ritonavir group (400 mg and 100 mg, respectively) and 100 patients to a standard-care group, and found no difference in viral clearance, time to clinical improvement, or 28-day mortality between the two groups. However, the trial was underpowered to exclude clinically relevant treatment effects.

**Added value of this study**

To our knowledge, the randomised evaluation of COVID-19 therapy (RECOVERY) trial is the first large-scale randomised clinical trial to report the effects of lopinavir–ritonavir in patients admitted to hospital with COVID-19. We found no significant difference between the lopinavir–ritonavir group and the usual care group in terms of 28-day mortality, the probability of discharge alive within 28 days, or, among patients who were not receiving invasive mechanical ventilation at randomisation, the probability of progressing to the composite outcome of invasive mechanical ventilation or death. We saw no evidence of benefit of lopinavir–ritonavir in any patient subgroup.

**Implications of all the available evidence**

Our finding of no clinical benefit from lopinavir–ritonavir treatment compared with standard care supports earlier findings from a smaller clinical trial. Many clinical care guidelines have recommended lopinavir–ritonavir for treatment of patients admitted to hospital with COVID-19. These guidelines should be updated.

## Methods

### Study design and participants

The randomised evaluation of COVID-19 therapy (RECOVERY) trial is an investigator-initiated, individually randomised, open-label, platform trial to evaluate the effects of potential treatments in patients admitted to hospital with COVID-19.^19^, ^20^ The trial is underway at 176 hospitals in the UK (appendix pp 2–20), supported by the National Institute for Health Research Clinical Research Network. The trial is coordinated by the Nuffield Department of Population Health at University of Oxford (Oxford, UK), the trial sponsor. The trial is done in accordance with the principles of the International Conference on Harmonisation–Good Clinical Practice guidelines and approved by the UK Medicines and Healthcare products Regulatory Agency and the Cambridge East Research Ethics Committee (20/EE/0101). The protocol, statistical analysis plan, and additional information are available on the study website.

Although the lopinavir–ritonavir, dexamethasone, and hydroxychloroquine groups have now been stopped, the trial continues to study the effects of azithromycin, tocilizumab, convalescent plasma, and REGN-CoV2 (a combination of two monoclonal antibodies directed against SARS-CoV-2 spike protein). Other treatments might be studied in the future.

Patients admitted to hospital were eligible for the study if they had clinically suspected or laboratory confirmed SARS-CoV-2 infection and no medical history that might, in the opinion of the attending clinician, put the patient at substantial risk if they were to participate in the trial. Initially, recruitment was limited to patients who were aged at least 18 years, but from May 9, 2020, this age limit was removed. Patients with severe hepatic insufficiency or who were using medicinal products that are highly dependent on cytochrome P450 3A4 for clearance and for whom elevated plasma concentrations would be associated with serious or life-threatening events (in line with the summary of product characteristics) were excluded from entry into the randomised comparison between lopinavir–ritonavir and usual care.^21^ Written informed consent was obtained from all patients, or their legal representative if they were too unwell or unable to provide consent.

### Randomisation and masking

Baseline data were collected using a web-based case report form that included demographics, amount of respiratory support, major comorbidities, suitability of the study treatments for a particular patient, and treatment availability at the study site (appendix p 25). Eligible and consenting patients were assigned to either usual standard of care or usual standard of care plus lopinavir–ritonavir or one of the other available RECOVERY treatment groups using web-based simple (unstratified) randomisation with allocation concealed until after randomisation. Randomisation to usual care was twice that of any of the active treatment groups the patient was eligible for (eg, 2:1 in favour of usual care if the patient was eligible for only one active group, 2:1:1 if the patient was eligible for two active groups). For some patients, lopinavir–ritonavir was unavailable at the hospital at the time of enrolment or was considered by the attending clinician to be either definitely indicated or definitely contraindicated. These patients were excluded from the randomised comparison between lopinavir–ritonavir and usual care and hence are not included in this report. Patients allocated to lopinavir–ritonavir were to receive lopinavir 400 mg plus ritonavir 100 mg by mouth every 12 h for 10 days or until discharge, if sooner. Allocated treatment was prescribed by the attending clinician. Participants and local study staff were not masked to the allocated treatment. The trial steering committee, investigators, and all other individuals involved in the trial were masked to outcome data during the trial.

### Procedures

A single online follow-up form was completed when participants were discharged alive from hospital, died, or at 28 days after randomisation, whichever occurred earliest (appendix pp 27–30). Information was recorded on adherence to allocated study treatment, receipt of other COVID-19 treatments, duration of admission, receipt of respiratory or renal support, and vital status (including cause of death). Additionally, routine health-care and registry data were obtained including information on vital status (with date and cause of death), discharge from hospital, receipt of respiratory support, or renal replacement therapy.

### Outcomes

Outcomes were assessed at 28 days after randomisation, with further analyses specified at 6 months. The primary outcome was 28-day all-cause mortality. Secondary outcomes were time to discharge from hospital and, among patients not on invasive mechanical ventilation at randomisation, post-enrolment use of invasive mechanical ventilation (including extracorporeal membrane oxygenation) or death. Prespecified subsidiary clinical outcomes were cause-specific mortality, use of haemodialysis or haemofiltration, major cardiac arrhythmia (recorded in a subset), and receipt and duration of ventilation (for which full data are still being collected from relevant routine sources).

Information on suspected serious adverse reactions was collected in an expedited fashion to comply with regulatory requirements.

### Statistical analysis

An intention-to-treat comparison was made between patients randomly assigned to lopinavir–ritonavir and patients randomly assigned to usual care but for whom lopinavir–ritonavir was both available and suitable as a treatment. For the primary outcome of 28-day mortality, the log-rank observed minus expected statistic and its variance were used to both test the null hypothesis of equal survival curves (ie, the log-rank test) and to calculate the one-step estimate of the average mortality rate ratio and its 95% CI. We constructed Kaplan-Meier survival curves to display cumulative mortality over the 28-day period. We used the same method to analyse time to hospital discharge, with patients who died in hospital right-censored on day 29 (as for such patients it was known that they were not discharged alive within 28 days). Median time to discharge was derived from Kaplan-Meier estimates. For the prespecified composite secondary outcome of invasive mechanical ventilation or death within 28 days (among those not receiving invasive mechanical ventilation at randomisation), the precise date of invasive mechanical ventilation was not available and so the risk ratio was estimated instead.

Prespecified analyses of the primary outcome were done separately in six subgroups defined by characteristics at the time of random assignment: age, sex, ethnicity, level of respiratory support, days since symptom onset, and predicted 28-day mortality risk (appendix p 23). Observed effects within subgroup categories were compared using a χ^2^ test for heterogeneity or trend, in accordance with the prespecified analysis plan.

Estimates of rate ratios and risk ratios are shown with 95% CIs. All p values are two-sided and are shown without adjustment for multiple testing. The full database is held by the study team that collected the data from study sites and did the analyses at the Nuffield Department of Population Health (University of Oxford, Oxford, UK).

As stated in the protocol, appropriate sample sizes could not be estimated when the trial was being planned since the numbers that could be enrolled are dependent on how large the epidemic becomes. However, our aim was to randomise several thousand patients (appendix p 22). The independent data monitoring committee reviewed unblinded analyses of the study data and any other information considered relevant at intervals of around 2 weeks. The committee was charged with determining if, in their view, the randomised comparisons in the study provided evidence on mortality that was strong enough (with a range of uncertainty around the results that was narrow enough) to affect national and global treatment strategies (appendix p 31). In such a circumstance, the committee would inform the chief investigators who would make the results available to the public and amend the trial accordingly.

On June 25, 2020, the independent data monitoring committee conducted a routine review of the data and recommended that the chief investigators review the unblinded data on the lopinavir–ritonavir comparison with usual care. The chief investigators and steering committee concluded that the data showed no beneficial effect of lopinavir–ritonavir in patients admitted to hospital with COVID-19. Therefore, enrolment of participants to the lopinavir–ritonavir group was closed on June 29, and the preliminary result for the primary outcome was made public. Investigators were advised that any patients currently taking lopinavir–ritonavir as part of the study should discontinue.

Analyses were done using Stata version 15.1 and R version 3.4 and validated using SAS version 4. The trial is registered with ISRCTN, 50189673, and ClinicalTrials.gov, NCT04381936.

### Role of the funding source

The funders of the study had no role in study design, data collection, data analysis, data interpretation, or writing of the report. PWH and MJL had full access to all the data in the study and had final responsibility for the decision to submit for publication.

## Results

**Figure 1:**
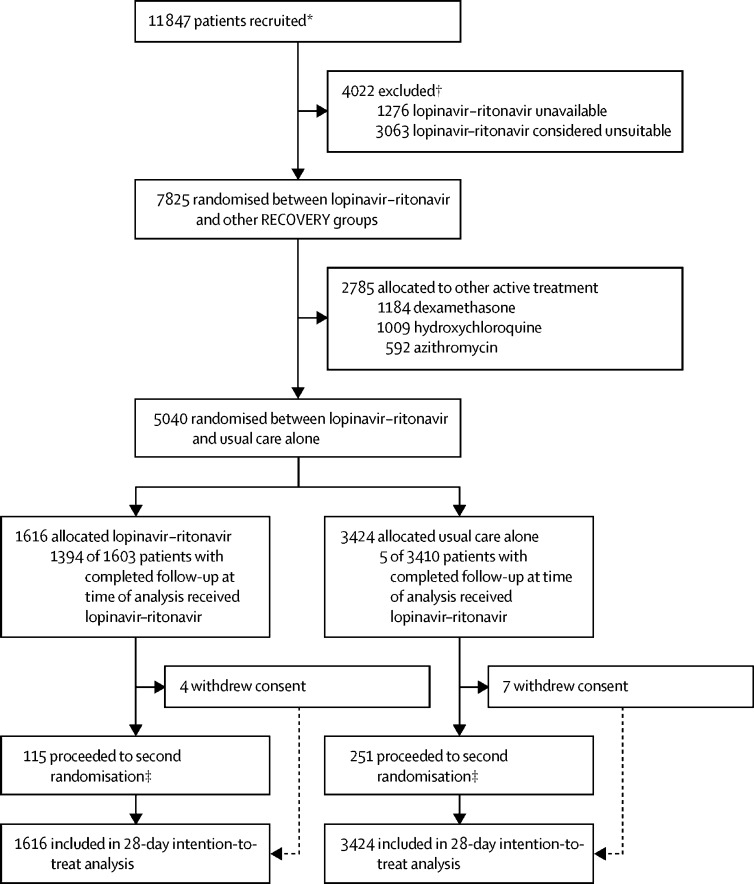
Trial profile *Number recruited overall during the period that participants could be recruited into lopinavir–ritonavir comparison. †Some patients were included in both of the below groups. ‡A second randomisation to tocilizumab versus usual care in patients with hypoxia and C-reactive protein ≥75 mg/L was introduced in protocol version 4.0.

Between March 19, 2020, and June 29, 2020, 7825 (66%) of 11 847 patients randomly assigned when the lopinavir–ritonavir group was open for enrolment were eligible to be randomly allocated to lopinavir–ritonavir (lopinavir–ritonavir was available in the hospital and the attending clinician was of the opinion that the patient had no known indication for or contraindication to lopinavir–ritonavir; figure 1; appendix p 33). 1616 patients were randomly allocated to lopinavir–ritonavir and 3424 were randomly allocated to usual care, with the remainder being randomly allocated to one of the other RECOVERY treatment groups. The mean age of study participants in this comparison was 66·2 years (SD 15·9; table 1; appendix p 33). At randomisation, about a quarter of patients had no ventilatory support, most were receiving oxygen only, and a very small proportion were on invasive mechanical ventilation (table 1).

**Table 1:** Baseline characteristics

		**Lopinavir–ritonavir (n=1616)**	**Usual care (n=3424)**
Age, years		66·0 (16·0)	66·4 (15·8)
	<70*	920 (57%)	1910 (56%)
	≥70 to <80	321 (20%)	706 (21%)
	≥80	375 (23%)	808 (24%)
Sex			
	Men	973 (60%)	2104 (61%)
	Women†	643 (40%)	1320 (39%)
Ethnicity			
	White	1240 (77%)	2541 (74%)
	Black, Asian, and minority ethnic	250 (15%)	615 (18%)
	Unknown	126 (8%)	268 (8%)
Number of days since symptom onset		8 (5–12)	8 (4–12)
Number of days since admission to hospital		2 (1–4)	2 (1–4)
Respiratory support received			
	No oxygen received	425 (26%)	896 (26%)
	Oxygen only‡	1131 (70%)	2384 (70%)
	Invasive mechanical ventilation	60 (4%)	144 (4%)
Previous diseases			
	Diabetes	430 (27%)	958 (28%)
	Heart disease	403 (25%)	908 (27%)
	Chronic lung disease	386 (24%)	776 (23%)
	Tuberculosis	4 (<1%)	12 (<1%)
	HIV	3 (<1%)	3 (<1%)
	Severe liver disease§	0	0
	Severe kidney impairment¶	113 (7%)	263 (8%)
	Any of the above	918 (57%)	1962 (57%)
Severe acute respiratory syndrome coronavirus 2 test result			
	Positive	1399 (87%)	3024 (88%)
	Negative	207 (13%)	388 (11%)
	Unknown	10 (1%)	12 (<1%)

Data are mean (SD), n (%), or median (IQR).

* Includes two children (<18 years).

† Includes six pregnant women.

‡ Includes non-invasive ventilation.

§ Defined as requiring ongoing specialist care.

¶ Defined as estimated glomerular filtration rate <30 mL/min per 1·73 m^2^.

Follow-up information was complete for 5018 (>99%) of 5040 patients (1606 [99%] of 1616 patients in the lopinavir–ritonavir group and 3412 [>99%] of 3424 patients in the usual care group). Among patients with a completed follow-up form, 1394 (87%) allocated to lopinavir–ritonavir received at least one dose (figure 1; appendix p 34). The median duration of treatment was 5 days (IQR 2–8). In the usual care group, five patients (<1%) received lopinavir–ritonavir. Use of azithromycin or another macrolide as part of clinical care during follow-up was similar in both groups (374 [23%] patients in the lopinavir–ritonavir group *vs* 862 [25%] in the usual care group), as was use of dexamethasone (160 [10%] *vs* 355 [10%]; appendix p 34).

51 (3%) of 1616 patients in the lopinavir–ritonavir group and 128 (4%) of 3424 patients in the usual care group proceeded to second randomisation and were allocated to tocilizumab in accordance with protocol version 4.0 or later. 72 patients were additionally randomly assigned to convalescent plasma versus control (19 [1%] patients allocated to lopinavir–ritonavir *vs* 53 [2%] patients allocated to usual care) in accordance with protocol version 6.0. Among the 163 sites that randomly assigned at least one patient to the lopinavir–ritonavir comparison, the median number of patients randomised was 22 (IQR 11–42).

**Figure 2:**
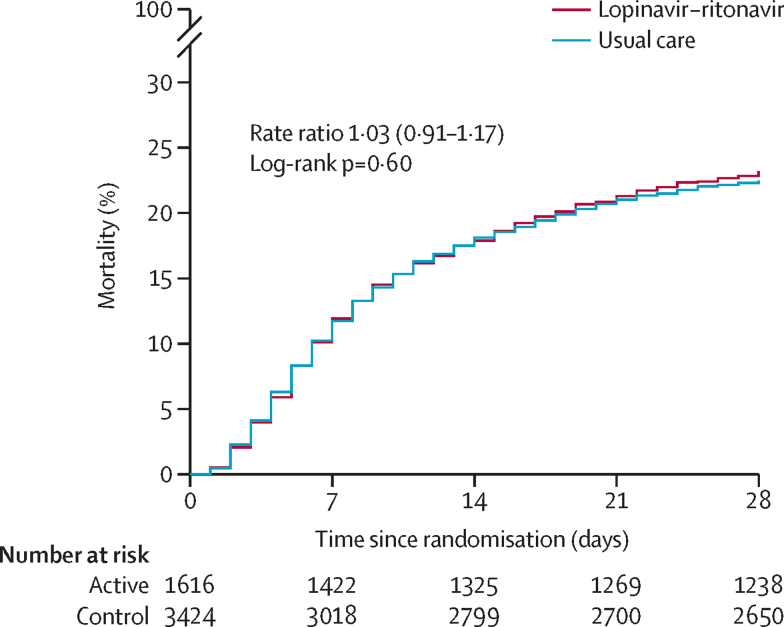
Effect of allocation to lopinavir–ritonavir on 28-day mortality

**Figure 3:**
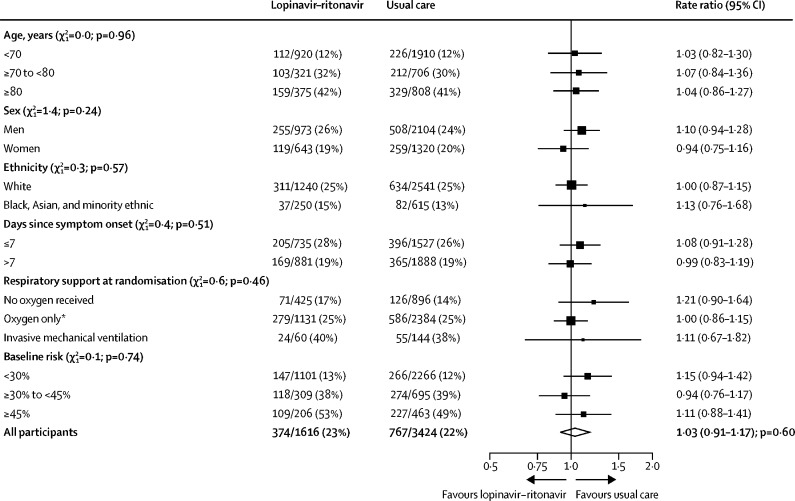
Effect of allocation to lopinavir–ritonavir on 28-day mortality by baseline characteristics Subgroup-specific rate ratio estimates are represented by squares (with areas of the squares proportional to the amount of statistical information) and the lines through them correspond to the 95% CIs. The ethnicity and days since onset subgroups exclude those with missing data, but these patients are included in the overall summary diamond. The method used to calculate baseline predicted risk is described in the appendix (p 23)). The χ^2^ statistics correspond to tests for trend (or heterogeneity) in the log rate ratios across the levels of each subgroup. *Includes patients receiving non-invasive ventilation.

We observed no significant difference in the proportion of patients who met the primary outcome of 28-day mortality between the two randomised groups (374 [23%] patients in the lopinavir–ritonavir group *vs* 767 [22%] patients in the usual care group; rate ratio 1·03, 95% CI 0·91–1·17; p=0·60; figure 2). We observed similar results across all prespecified subgroups (figure 3). In a post-hoc exploratory analysis restricted to the 4423 (88%) patients with a positive SARS-CoV-2 test result, the result was similar (rate ratio 1·05, 0·92–1·19; p=0·49).

Allocation to lopinavir–ritonavir was associated with a similar time until discharge alive from hospital as usual care (median 11 days [IQR 5 to >28] in both groups) and a similar probability of discharge alive from hospital within 28 days (rate ratio 0·98, 95% CI 0·91–1·05; p=0·53; table 2). Among individuals not on invasive mechanical ventilation at baseline, the number of patients who progressed to the prespecified composite secondary outcome of invasive mechanical ventilation or death among those allocated to lopinavir–ritonavir was similar to that among those allocated to usual care (risk ratio 1·09, 0·99–1·20; p=0·092; table 2).

**Table 2:** Effect of allocation to lopinavir–ritonavir on key study outcomes

		**Lopinavir–ritonavir (n=1616)**	**Usual care (n=3424)**	**RR (95% CI)**	**p value**
**Primary outcome**					
28-day mortality		374 (23%)	767 (22%)	1·03 (0·91–1·17)	0·60
**Secondary outcomes**					
Discharged from hospital within 28 days		1113 (69%)	2382 (70%)	0·98 (0·91–1·05)	0·53
Receipt of invasive mechanical ventilation or death*		449/1556 (29%)	871/3280 (27%)	1·09 (0·99–1·20)	0·092
	Invasive mechanical ventilation	152/1556 (10%)	279/3280 (9%)	1·15 (0·95–1·39)	0·15
	Death	350/1556 (22%)	712/3280 (22%)	1·04 (0·93–1·16)	0·54

Data are n (%) or n/N (%), unless otherwise indicated. RR=rate ratio for the outcomes of 28-day mortality and hospital discharge, and risk ratio for the outcome of receipt of invasive mechanical ventilation or death (and its subcomponents).

* Analyses exclude those on invasive mechanical ventilation at randomisation.

We found no significant differences in cause-specific mortality (appendix p 35). Among those not on renal dialysis or haemofiltration at randomisation, the number of patients requiring this treatment within 28 days was similar between the two groups (66 [4%] of 1588 patients in the lopinavir–ritonavir group *vs* 140 [4%] of 3348 patients in the usual care group; risk ratio 0·99, 95% CI 0·75–1·32; p=0·97). We observed no significant differences in the frequency of new cardiac arrhythmias (appendix p 36). There was one report of a serious adverse reaction thought to be related to lopinavir–ritonavir, which was a case of elevated alanine aminotransferase that did not meet standard criteria for drug-induced liver injury and from which the patient recovered after stopping treatment.

## Discussion

The results of this large randomised trial indicate that lopinavir–ritonavir is not an effective treatment for patients admitted to hospital with COVID-19. The lower bound of the confidence limit for the primary outcome rules out any reasonable possibility of a meaningful mortality benefit. Additionally, allocation to lopinavir–ritonavir was not associated with reductions in the duration of hospital stay or the risk of being ventilated or dying for those not on ventilation at baseline. These results were consistent across subgroups of age, sex, ethnicity, duration of symptoms before randomisation, amount of respiratory support at randomisation, and baseline predicted risk of death at randomisation.

It is unclear whether the dose of lopinavir–ritonavir we used achieved adequate SARS-CoV-2 inhibitory concentrations in the lungs.^22^ Data on the in vitro 50% maximum effective concentration (EC_50_) of lopinavir–ritonavir against SARS-CoV-2 are limited and variable, having been variously reported as 3·6, 5·7, and 9·6 μg/mL.^23^ Although the extensive protein binding of lopinavir (>95%) might result in unbound plasma drug concentrations below the highest reported EC_50_,^22^, ^24^ the reported in vitro EC_50_ data have been generated in the presence of some serum protein and so do not represent a zero protein binding value. Additionally, concentrations of lopinavir in patients with COVID-19 have been reported to be substantially higher than in patients with HIV, perhaps due to inhibition of CYP3A4 metabolism by inflammation.^24^, ^25^, ^26^, ^27^ A pharmacokinetic analysis of a range of putative SARS-CoV-2 antiviral drugs has predicted that lopinavir–ritonavir at the doses we used would achieve lung concentrations above the EC_90_, albeit not across the entire dosing interval.^23^

Our trial, which focused on providing clear information on the effect of lopinavir–ritonavir on unambiguous clinical outcomes, including all-cause mortality, has several limitations. Since the safety profile of lopinavir–ritonavir is well established, we did not collect detailed information on non-serious adverse reactions or reasons for stopping treatment. Neither did we collect information on physiological, laboratory, or virological parameters, which have been studied previously.^17^ Finally, very few intubated patients with COVID-19 were enrolled in this study as there were difficulties in administering treatment to patients who could not swallow. Crushing lopinavir–ritonavir tablets for administration down a feeding tube results in unreliable bioavailability and potential tube blockage.^28^, ^29^ Although a liquid formulation of lopinavir–ritonavir exists, it was unavailable for this study and, since the liquid formulation contains alcohol, is not compatible with the polyurethane feeding tubes that are commonly used in the UK.

The results from the RECOVERY trial show that lopinavir–ritonavir monotherapy is not an effective treatment for patients admitted to hospital with COVID–19. Treatment of COVID-19 with lopinavir–ritonavir has been recommended as a first-line or second-line in many countries.^3^ Since our preliminary results were made public on June 29, 2020, WHO has halted the lopinavir–ritonavir monotherapy and the lopinavir–ritonavir plus interferon beta combination groups of the SOLIDARITY trial because the interim results are in line with those presented here—lopinavir–ritonavir does not improve clinical outcomes for patients admitted to hospital with COVID-19.^30^

## Data sharing

The protocol, consent form, statistical analysis plan, definition and derivation of clinical characteristics and outcomes, training materials, regulatory documents, and other relevant study materials are available online at www.recoverytrial.net. As described in the protocol, the trial steering committee will facilitate use of the study data and approval will not be unreasonably withheld. Deidentified participant data will be made available to bona fide researchers registered with an appropriate institution within 3 months of publication. However, the steering committee will need to be satisfied that any proposed publication is of high quality, honours the commitments made to the study participants in the consent documentation and ethical approvals, and is compliant with relevant legal and regulatory requirements (eg, relating to data protection and privacy). The steering committee will have the right to review and comment on any draft manuscripts before publication. Data will be made available in line with the policy and procedures described at https://www.ndph.ox.ac.uk/data-access. Those wishing to request access should complete the form at https://www.ndph.ox.ac.uk/files/about/data_access_enquiry_form_13_6_2019.docx and e-mail data.access@ndph.ox.ac.uk.
