# Supplementary material for: Lopinavir–ritonavir in patients admitted to hospital with COVID-19 (RECOVERY): a randomised, controlled, open-label, platform trial
Source: Lancet. 2020 Oct 24;396(10259):1345–52. doi: 10.1016/S0140-6736(20)32013-4 (PMC7535623; doi:10.1016/S0140-6736(20)32013-4)
Supplement: Supplementary appendix [file mmc1.pdf]

THE LANCET

Supplementary appendix

This appendix formed part of the original submission and has been peer reviewed. We post it as supplied by the authors.

Supplement to: RECOVERY Collaborative Group. Lopinavir–ritonavir in patients admitted to hospital with COVID-19 (RECOVERY): a randomised, controlled, open-label, platform trial. *Lancet* 2020; published online Oct 5. http://dx.doi.org/10.1016/S0140-6736(20)32013-4.

Lopinavir-ritonavir in Hospitalised Patients with COVID-19 – a randomised, controlled, open-label, platform trial

SUPPLEMENTARY APPENDIX

RECOVERY Collaborative Group

Contents

Details of the RECOVERY Collaborative Group 2
Supplementary Methods... 21
Study organization 21
Protocol changes 21
Supplementary statistical methods 22
Sample size.. 22
Baseline-predicted risk 23
Ascertainment and classification of study outcomes 23
Randomisation form 23
Follow-up form... 26
Interim analyses: role of the Data Monitoring Committee 31
Supplementary Tables 32
Webtable 1: Baseline characteristics of patients considered unsuitable for randomisation to lopinavir-ritonavir compared with those randomised to lopinavir-ritonavir versus usual care 33
Webtable 2: Treatments given, by randomized allocation 34
Webtable 3: Effect of allocation to lopinavir-ritonavir on cause-specific 28-day mortality 35
Webtable 4: Effect of allocation to lopinavir-ritonavir on cardiac arrhythmia 36

Details of the RECOVERY Collaborative Group

Writing Committee

Professor Peter W Horby FRCP,[a,*] Marion Mafham MD,[b,*] Jennifer L Bell MSc,[b,*] Louise Linsell DPhil,[b] Natalie Staplin PhD,[b,c] Professor Jonathan Emberson PhD,[b,c] Adrian Palfreeman FRCP,[d] Jason Raw FRCP,[e] Einas Elmahi MPhil,[f] Benjamin Prudon FRCP,[g] Christopher Green DPhil,[h] Professor Simon Carley MD,[i] David Chadwick PhD,[j] Matthew Davies MB ChB,[k] Matthew P Wise DPhil,[l] J Kenneth Baillie MD PhD,[m] Professor Lucy C Chappell PhD,[n] Professor Saul N Faust FRCPCH,[o] Professor Thomas Jaki PhD,[p,q] Katie Jeffery PhD,[r] Professor Wei Shen Lim FRCP,[s] Professor Alan Montgomery PhD,[t] Kathryn Rowan PhD,[u] Professor Edmund Juszczak MSc,[b,t,†] Professor Richard Haynes DM,[b,c,†] Professor Martin J Landray PhD.[b,c,v,†]

[a] Nuffield Department of Medicine, University of Oxford, Oxford, United Kingdom.
[b] Nuffield Department of Population Health, University of Oxford, Oxford, United Kingdom
[c] MRC Population Health Research Unit, University of Oxford, Oxford, United Kingdom
[d] Department of Infectious Diseases, University Hospital Leicester, Leicester, United Kingdom
[e] Fairfield General Hospital, Pennine Acute Hospitals NHS Trust, Bury, United Kingdom
[f] Research and Development Department, Northampton General Hospital, Northampton, United Kingdom
[g] Department of Respiratory Medicine, North Tees & Hartlepool NHS Foundation Trust, Stockton-on-Tees, United Kingdom
[h] University Hospitals Birmingham NHS Foundation Trust and Institute of Microbiology & Infection, University of Birmingham, Birmingham, United Kingdom
[i] Manchester Metropolitan University, and Manchester University NHS Foundation Trust, Manchester, United Kingdom
[j] Centre for Clinical Infection, James Cook University Hospital, Middlesbrough, United Kingdom
[k] North West Anglia NHS Foundation Trust, Peterborough, United Kingdom
[l] Adult Critical Care, University Hospital of Wales, Cardiff, United Kingdom
[m] Roslin Institute, University of Edinburgh, Edinburgh, United Kingdom
[n] School of Life Course Sciences, King's College London, London, United Kingdom
[o] NIHR Southampton Clinical Research Facility and Biomedical Research Centre, University Hospital Southampton NHS Foundation Trust and University of Southampton, Southampton, United Kingdom
[p] Department of Mathematics and Statistics, Lancaster University, Lancaster, United Kingdom
[q] MRC Biostatistics Unit, University of Cambridge, Cambridge, United Kingdom
[r] Oxford University Hospitals NHS Foundation Trust, Oxford, United Kingdom
[s] Respiratory Medicine Department, Nottingham University Hospitals NHS Trust, Nottingham, United Kingdom
[t] Nottingham Clinical Trials Unit, University of Nottingham, Nottingham, United Kingdom
[u] Intensive Care National Audit & Research Centre, London, United Kingdom
[v] NIHR Oxford Biomedical Research Centre, Oxford University Hospitals NHS Foundation Trust, Oxford, United Kingdom
*,† equal contribution

Steering Committee

Co-Chief Investigators PW Horby, MJ Landray; *Clinical Trial Unit Leads* R Haynes, E Juszczak; *Members* JK Baillie, L Chappell, SN Faust, T Jaki, K Jeffery, WS Lim, M Mafham, A Montgomery, K Rowan.

Data Monitoring Committee

P Sandercock (chair), J Darbyshire, D DeMets, R Fowler, D Lalloo, I Roberts, J Wittes *Non-voting statisticians* J Emberson, N Staplin.

RECOVERY Trial Central Coordinating Office

Co-Chief Investigators P Horby, MJ Landray; *Clinical Trial Unit Leads* R Haynes, E Juszczak; *Trial management* L Fletcher (coordinator), J Barton, A Basoglu, R Brown, W Brudlo, S Howard, K Taylor; *Programming and validation* B Goodenough, G Cui, A King, M Lay, D Murray, W Stevens, K Wallendszus, R Welsh; *Data linkage* C Crichton, J Davies, R Goldacre, F Knight, J Latham-Mollart, M Mafham, M Nunn, H Salih, J Welch; *Clinical support* G Pessoa-Amorim; *Quality assurance* C Knott, J Wiles; *Statistics* JL Bell, J Emberson, E Juszczak, L Linsell, N Staplin; *Communications* G Bagley, S Cameron, S Chamberlain, B Farrell, H Freeman, A Kennedy, A Whitehouse

National Institute for Health Research Clinical Research Network

Coordinating Centre A Barnard, J Beety, C Birch, M Brend, E Chambers, L Chappell, S Crawshaw, C Drake, H Duckles-Leech, J Graham, T Harman, H Harper, S Lock, K Lomme, N McMillan, I Nickson, U Ohia, E OKell, V Poustie, S Sam, P Sharratt, J Sheffield, H Slade, W Van't Hoff, S Walker, J Williamson; *Urgent Public Health Clinical Links* A De Soyza, P Dimitri, SN Faust, N Lemoine, J Minton; *East Midlands* K Gilmour, K Pearson *Eastern* C Armah, D Campbell, H Cate, A Priest, E Thomas, R Usher; *North East & North Cumbria* G Johnson, S Pratt, A Price, K Shirley, P Williams, F Yelnoorkar; *Kent, Surrey & Sussex* J Hanson, H Membrey, L Gill, A Oliver; *North West London* S Das, S Murphy, M Sutu; *Greater Manchester* J Collins, H Monaghan, A Unsworth, S Beddows; *North West Coast* S Dowling, K Gibbons, K Pine; *North Thames* A Asghar, P Aubrey, D Beaumont-Jewell, K Donaldson, T Skinner; *South London* J Luo, N Mguni, N Muzangi, R Pleass, E Wayman; *South West Peninsula* A Coe, J Hicks, M Hough, C Levett, A Potter, J Taylor; *Thames Valley and South Midlands* M Dolman, L Gerdes, C Hall, T Lockett, D Porter *Wessex* L Dowden, J Bartholomew, C Rook, J Walters; *West of England* E Denton, H Tinkler; *Yorkshire & Humber* A Alexander, H Campbell, K Chapman, A Hall, A Rodgers; *West Midlands* P Boyle, C Callens, H Duffy, C Green, K Hampshire, S Harrison, J Kirk, M Naz, L Porter, P Ryan, J Shenton, J Warmington; *Devolved nations* M Amezaga, P Dicks, J Goodwin, S Jackson, M Odam, D Williamson.

Paediatric working group

SN Faust (coordinator), A Bamford, J Bernatoniene, K Cathie, P Dmitri, S Drysdale, A Finn, P Fleming, J Furness, C Gale, R Haynes, CE Jones, E Juszczak, C Murray, N Pathan, A Ramanan, J Standing, C Roehr, M Wan, E Whittaker.

Obstetric working group

L Chappell (coordinator), M Knight, S Pavord, C Williamson.

Clinical support

NHS Lothian Out of Hours support line team M Odam (coordinator), P Black, B Gallagher, L MacInnes, R O'Brien, K Priestley, A Saunderson; *Clinical Trial Service Unit Out of Hours clinical support* L Bowman, F Chen, R Clarke, M Goonasekara, R Haynes, W Herrington, P Judge, M Mafham, S Ng, D Preiss, C Reith, E Sammons, D Zhu.

Health records

NHS DigiTrials, Southport H Pinches, P Bowker, V Byrne-Watts, G Chapman, G Coleman, J Gray, C Hooper, A Rees, MJ Landray, M Mafham, N Mather, T Denwood; *Intensive Care National Audit & Research Centre, London* D Harrison; *National Records of Scotland* G Turner; *Public Health Scotland* J Bruce; *SAIL Databank, University of Swansea* C Arkley, S Rees.

Drug supply

Public Health England and Department of Health and Social Care (DHSC) Vaccines & Countermeasures teams, DHSC Medicines Supply Contingency Planning Team, NHS England, NHS Improvement, Movianto UK Ltd, Supply Chain Coordination Ltd.

Local Clinical Centre RECOVERY trial staff

(listed in descending order of the number of patients randomised per site)

University Hospitals Of Leicester NHS Trust C Brightling (PI), N Brunskill (Co-PI), M Wiselka (Co-PI), S Bandi, S Batham, T Beaver, K Bhandal, M Bourne, L Boyles, A Charalambou, CK Cheung, R Cotter, S Diver, A Dunphy, O Elneima, J Fawke, J Finch, C Gardiner-Hill, G Genato, M Graham-Brown, C Haines, B Hargadon, H Holdsworth, W Ibrahim, L Ingram, JA Jesus Silva, K Kaul, A Kuverji, K-T Kyriaki, A Lea, T Lee, L Lock, R Major, H McAuley, P McCourt, D Mullasseril Kutten, A Palfreeman, E Parker, M Patterson, L Plummer, D Samuel, H Selvaskandan, SM Southin, KK Tsilimpari, C Wiesender, A Yousuf.

Pennine Acute Hospitals NHS Trust A Ustianowski (PI), J Raw (Co-PI), R Tully (Co-PI), Z Antonina, E Ayaz, P Bradley, F Bray, C Carty, G Connolly, C Corbett, S Dermody, L Durrans, E Falconer, J Flaherty, D Hadfield, L Hoggett, A Horsley, S Hussain, R Irving, P Jacob, D Johnstone, R Joseph, P Kamath, T Khatun, T Lamb, H Law, G Lindergard, S Lokanathan, L Macfarlane, S Mathen, S McCullough, P McMaster, D McSorland, J Melville, B Mishra, S Munt, A Neal, R Newport, G O'Connor, D O'Riordan, I Page, V Parambil,J Philbin, C Rishton, M Riste, M Sam, Z Sarwar, L Scarratt, H Sharaf, J Shaw, J Shaw, A Slack, A Uriel, O Walton.

Nottingham University Hospitals NHS Trust WS Lim (PI), A Andrews, L Anderson, D Ashton, G Babington, G Bartlett, D Batra, L Bendall, T Brear, A Buck, G Bugg, J Butler, J Cantliff, L Clark, P Davies, M Dent, A Fatemi, M Fatemi, L Hodgen, S Hodgson, S Hodgkinson, C Hutchinson, B Jackson, E Keddie-Gray, C Khurana, M Langley, M Meredith, L Morris, H

Navarra, B Petrova, C Peters, Z Rose, L Ryan, J Sampson, G Squires, R Taylor, J Thornton, S Warburton, S Wardle, S Wei, T Wildsmith, L Wilson.

Northampton General Hospital NHS Trust E Elmahi (PI), M Zaman (Co-I), B Abdul, A Abdulmumeen, MH Ahammed Nazeer, A Bazli, N Benesh, N Cunningham, H Daggett, E Davies, H Enyi, S Fawohunre, N Geoghegan, J Glover, K Hall, K Haresh, WU Hassan, J Hosea, M Idrees, C Igwe, H Imtiaz, M Irshad, A Ismail, R Jeffrey, J Jith, P Joshi, R Kaliannan Periyasami, A Khalid, MU Khalid, R Kodituwakku, P Lopez, A Mahmood, M Malanca, VK Maruthamuthu, S Masood, F Merchant, N Natarajan, R Natarajan, O Ndefo, O Ogunkeye, S Paranamana, N Pugh, A Raj, K Rashid, M Rogers, M Saad, M Shahzeb, N Shrestha, A Singh, K Smith, B Sohail, M Spinks, L Stockham, A Takyi, YH Teoh, H Vayalaman, SEI Wafa, T Ward, R Watson, R Watson, L Ylquimiche Melly.

North Tees and Hartlepool NHS Foundation Trust B Prudon (PI), N Aung (Co-PI), R Srinivasan (Co-PI), S Wild (Co-PI), C Adams, D Barker, B Campbell, V Collins, J Deane, S Gowans, L Poole, S Purvis, J Quigley, A Ramshaw, L Shepherd, J Skelton, R Taylor, M Walker, M Weetman, B Wetherall.

University Hospitals Birmingham NHS Foundation Trust C Green (PI), I Ahmed, N Anderson, C Armstrong, A Bamford, H Bancroft, M Bates, S Begum, M Bellamy, C Bergin, K Bhandal, E Brandl-Salutz, E Buckingham, E Burke, M Carmody, L Cooper, J Daglish, J Dasgin, A Desai, S Dhani, D Dosanjh, H Ellis, D Gardiner, E Grobovaite, B Hopkins, D Hull, J Jones, L Khan, D Lenton, M Lewis, M Lovell, F Lowe,
D Lynch, C McGhee, C McNeill, F Moore, A Nilsson, J Nunnick, C Prest, V Price, J Rhodes, J Sale, M Sangombe, H Smith, I Storey, L Thrasyvoulou, K Tsakiridou, D Walsh, S Welch, T Whitehouse, H Willis, J Woodford, G Wooldridge, C Zullo.

South Tees Hospitals NHS Foundation Trust D Chadwick (PI), S Armstrong, D Athorne, M Branch, S Brown, Y Chua, N Cunningham, J Dodds, S Dorgan, D Dunn, P Harper, H Harwood, K Hebbron, P Lambert, D Leaning, T Manders, C Milne, W Mohammad, A Murad, C Proctor, S Rao, MA Seelarbokus, P Singh, L Thompson, L Wiblin, J Williams, P Winder, C Wroe.

Manchester University NHS Foundation Trust T Felton (PI), T Abraham, S Akili, C Avram, M Baptist, R Bazaz, A Bikov, K Birchall, S Bokhari, G Calisti, S Carley, S Chilcott, C Chmiel, E Church, R Clark, H Dalgleish, A Desai, H Durrington, C Eades, G Evans, S Fowler, T Gorsuch, G Grana, G Gray, J Henry, A Horsley, L James, A John, E Johnstone, Z Kausar, A Khan, E Kolakaluri, C Kosmidis, RW Lord, L Manderson, G Margaritopoulos, C Mendonca, C Murray, R Norton, A Palacios, A Panes, L Peacock, S Ratcliffe, C Reynard, E Rice, P Rivera Ortega, A Simpson, J Soren, M Tin, R Tousis, R Wang, C Whitehead.

North West Anglia NHS Foundation Trust K Rege (PI), C Agbo, O Akindolie, A Al-Rabahi, R Ambrogetti, A Azman Shah, J Bhayani, T Bond, H Boughton, S Brooks, N Butterworth-Cowin, R Buttery, P Carter, L Cave, S Choi, N Duff, L Dufour, O Ebigbola, C Eddings, J Faccenda, P Goodyear, R Gooentilleke, R Gosling, W Halford, T Hoskins, C Huson, M Ishak, H Javed, T Jones, N Kasianczuk, D Kaur, A Kerr, A-I Khan, G Koshy, J Marshall, K McDevitt, T Okpala, T Old, G Oleszkiewicz, H Orme, S O'Sullivan, P Paczko, A Patel, S Pathak, S Poon, SHM Rizvi, M Samyraju, J Sanyal, E Smith, S Stacpoole, BT Tan, N Temple, K Thazhatheyil, MS Uddin.

Cardiff & Vale University LHB C Fegan (PI), A Balan, B Basker, S Bird, Z Boult, V Britten, H Cendl, J Cole, M Edger, M Evans, T Evans,F Greaves, S Harrhy, M Haynes, H Hill, Z Hilton, S Jorgensen, A Kelly, L Knibbs, D Lau, E Maureen, A McQueen, J Milner, R Norman, K Nyland, C Oliver, M Patal, K Rahilly, C Robinson, S Scourfield, M Starr, E Thomas, R Thomas-Turner, G Williams, M Williams, S Zaher.

Oxford University Hospitals NHS Foundation Trust K Jeffery (PI), M Ainsworth, C Arnison-Newgass, A Bashyal, S Beer, A Bloss, D Buttress, W Byrne, A Capp, P Carter, P Cicconi, R Corrigan, C Coston, L Cowen, N Davidson, L Downs, J Edwards, R Evans, D Georgiou, A Gillesen, A Harin, M Havinden-Williams, R Haynes, C Hird, A Hudak, P Hutton, R Irons, P Jastrzebska, S Johnston, M Kamfose, K Lewis, T Lockett, FM Maria del Rocio, JC Martinez Garrido, S Masih, A Mentzer, S Morris, C O'Callaghan, Z Oliver, E Perez, L Periyasamy, L Peto, D Porter, S Prasath, C Purdue, M Ramasamy, C Roehr, A Rudenko, V Sanchez, A Sarfatti, M Segovia, T Sewdin, J Seymour, V Skinner, L Smith, A Sobrino Diaz, M Taylor-Siddons, H Thraves, C Tsang, M Vatish, Y Warren, E Wilcock.

Luton and Dunstable University Hospital NHS Foundation Trust D Shaw (PI), S Tariq (Co-PI), N Ahmed, S Ali, S Allen, M Alzetani, C Ambrose, R Banerjee, T Baqai,A Batla, M Bergstrom, S Bhakta, T Chapman, A David, L Dirmantaite, T Dr.Angel, M Edmondson, H El-Sbahi, D Fishman, C Fornolles, T Forshall, A Francioni, S Gent, N George, A Ibrahim, A Ingram, R James, K Kabiru Dawa, F Khan, S Lee, C Lingam, N Marcus, M Masood, A Moharram, C Moss, G Naik, L Nicholls, M Nisar, V Parmar, F Prasanth Raj, V Quick, B Ramabhadran, A Reddy, N Riaz, B Rudran, S Sarma, K Savlani, P Shah, D Shaw, S-C Soo, P Sothirajah, I Southern, ML Tate, C Travill, W Wakeford.

Epsom and St Helier University Hospitals NHS Trust S Winn (PI), R Wake (Co-PI), S Ahamed Sadiq, A Aldana, B Al-Hakim, KA Agyapong, R Chicano, I Chukwulobelu, N Colbeck, N Cole, R Dogra, A Elradi, J Emberton, R Ganapathy, M Haque, R Hayre, S Jain, K Jian, A Johnson, L Johnson, J Kotecha, A Kundu, Y Mashhoudi, K Mathias, M-E Maxan, F Mellor, M Morgan, P Mysore, S Nafees, S Ramanna, J Ratoff, S Rozewicz, TDL Samuel, S Shahnazari, R Shail, A Sharif, S Somalanka, R Suckling, PA Swift, N Vilimiene, C Wells.

Buckinghamshire Healthcare NHS Trust R West (PI), J Abrams, A Baldwin, J Barker, H Blamey, E Chan, J Chaplin, B Chisnall, C Cleaver, S Crotty, P Dey, M Kononen, S Kudsk-Iversen, J Mandeville, S Mclure, A Ngumo, R Oxlade, M Rahman, C Robertson, S Shah, J Tebbutt, M Veres, N Wong, M Zammit-Mangion, M Zia.

Frimley Health NHS Foundation Trust M Meda (PI), J Democratis (PI), N Barnes, N Brooks, L Chapman, J da Rocha, R Dolman, S Gee, S Jaiswal, M Molloholli, F Regan, L Rowe-Leete, C Smith, M Van De Venne, T Weerasinghe.

NHS Lothian: Royal Infirmary of Edinburgh A Gray (PI), JK Baillie (Co-I), M Adam, A Anand, R Anderson, D Baird, T Balaskas, J Balfour, P Black, C Blackstock, R Campbell, P Chapman, C Cheyne, A Christides, D Christmas, L Crisp, D Cryans, J Dear, M Docherty, R Dodds, L Donald, M Eddleston, N Fethers, D Gilliland, E Godson, J Grahamslaw, S Hainey, M Harvey, D Henshall, S Hobson, N Hunter, K Htet Htet Ei, Y Jaly, J Jameson, D Japp, L Kitto, S Krupej, C Langoya, R Lawrie, A Lloyd, B Lyell, D Lynch, L MacInnes, A MacRaild, A Marshall, C McCann, F McCurrach, E Moatt, W Morley, M Morrissey, K Nizam Ud Din, R O'Brien, E O'Sullivan, M Odam, A Peterson, P Phelan, N Robertson, N Rowan, R Al-Shahi Salman, E Small, P Stefanowska, A Stevenson, S Stock, A Summers, J Teasdale, I Walker, K Walker, A Williams.

Wrightington, Wigan and Leigh NHS Foundation Trust A Ashish (PI), V Amit, J Cooper, D Heaton, V Parkinson, E Robinson, T Taylor, C Tierney, N Waddington, C Zipitis.

Barts Health NHS Trust S Tiberi (PI), A Aboaba, E Adeyeye, J Agwada-Akeru, FR Ali, C Ardley, R Astin-Chamberlain, G Bacon, H Baillie, R Batha, B Bloom, M Bolton, C Borra, G Boyapati, R Buchanan, C Chan, C Chitsenga, B Cipriano, P Foster Cofie, M DeLuna, K El-Shakankery, A Fikree, A Ghosh, R Goiriz, P Goldsmith,M Gouldbourne, A Grant, L Greenfield, S Grigoriadou, R Grittom, J Hand, C Harwood, U Hemmila, J Higgins, L Howaniec, D Hsu, S Issa, P Jones, M Juan, J Kassam, C Keith-Jopp, H Kunst, I Lee, D Lieberman, E Magavern,

C Maniero, J Maitland, N Matin, P May, R McDermott, K Menacho, L Millin, A Mohammed, K Moriarty, T Newman, C Nicfhogartaigh, A Pakozdi, M Parrott, P Pfeffer, J Pott, J Powell, W Ricketts, V Sarodaya, B Selvarajah, I Skene, A So, D Stevenson, S Thomas, J Thomson, N Thorn, C Tierney, S Ullah, R Vathenen, K Ward, P Woodland, S Youssouf, A Zdanaviciene.

Chesterfield Royal Hospital NHS Foundation Trust N Spittle (PI), N Weatherly (Co-PI), S Beavis, J Bradder, J Cort, J Cresswell, K Dale, A Foo, J Gardner, R Gascoyne, E Hall, M Kelly-Baxter, E Mackay, K Pritchard, J Salmon, A Smith, V Sorice, L Stevenson, A Whileman, E Wolodimeroff.

Dartford and Gravesham NHS Trust B Khan (PI), D Ail, R Aldouri, G Awadzi, R Bhalla, S Bokhari, G Boniface, J Cernova, T Chen, N Chitalia, S Danso-Bamfo, A Dhanoa, T Edmunds, E Fernandez, T Ferrari, B Fuller, A Gherman, R Heire, L Ilves, L Lacey, E Lawrence, M Lewis, A Maric, W Martin, Z Min, C Newman, R Nicholas, O Olufuwa, T Qadeer, S Rathore, S Sathianandan, A Shonubi, S Siddique, G Sisson, M Soan, D Streit, C Stuart, W Umeojiako, S Urruela, B Warner, M Waterstone, S White, K Yip, A-S Zafar, S Zaman.

Northumbria Healthcare NHS Foundation Trust B Yates (PI), C Ashbrook-Raby, H Campbell, D Charlton, V Ferguson, T Hall, I Hamoodi, P Heslop, J Luke, S Pick, J Reynolds, S Robinson, C Walker.

North Middlesex University Hospital NHS Trust J Moreno-Cuesta (PI), S Rokadiya (Co-PI), A Govind, A Haldeos, K Leigh-Ellis, V Rachel, C van Someren, R Vincent**.**

Countess Of Chester Hospital NHS Foundation Trust S Scott (PI), M Abouibrahim, M Ahmad, SH Ahmed, A Ajibode, L Alomari, E Austin, P Bamford, K Barker-Williams, W Barnsley, I Benton, S Billingham, S Brearey, S Brigham, V Brooker, C Burchett, K Cawley, Z Cheng, R Clarke, C Cotton, A Davidson, LN Ellerton, L Gamble, M Grant, J Grounds, H Hodgkins, M Iyer, A Johari, C Jones,
N Kearsley, B Lim, DK Llanera, E London, E Martin, P Maskell, M McCarthy, R McEwen, E Meeks, G Metcalf-Cuenca, S Middleton, L Mihalca-Mason, SU Rahman, S Scott, C Thorne, T Trussell, L Zammit.

Surrey and Sussex Healthcare NHS Trust E Potton (PI), N Jain (Sub-I), A Khadar (Sub-I), P Morgan (Sub-I), J Penny (Sub-I), E Tatam (Sub-I), S Abbasi, D Acharya, A Acosta, L Ahmed, S Ali, M Alkhusheh, V Amosun, A Arter, M Babi, J Bacon, K Bailey, N Balachandran, S Bandyopadhya, L Banks, J Barla, T Batty, S Bax, A Belgaumkar, G Benison-Horner, A Boles, N Broomhead, E Cetti, C Chan, I Chaudhry, D Chudgar, J Clark, S Clueit, S Collins, E Combes, G Conway, O Curtis, M Das, M Daschel, S Davies, A Day, M Dhar, K Diaz-Pratt, C Dragan, H Dube, V Duraiswamy, J Elias, A Ellis, T-Y Ellis, J Emmanuel, A Engden, Y Fahmay, B Field, K Fishwich, U Ganesh, C Gilbert, E Goudie, S Griffith, S Gurung, R Habibi, C Halevy, A Haqiqi, R Hartley, A Hayman, J Hives, M Horsford, S Hughes, C Hui, R Hussain, C Iles, L Jackson, A James, D Jayaram, E Jessup-Dunton, T Joefield, N Khan, W Kieffer, E Knox, V Kumar, R Kumar, V Kurmars, H Lafferty, F Lamb, R Layug, N Leitch, W Lim, U Limbu, R Loveless, M Mackenzie, N Maghsoodi, S Maher, M Maljk, I Man, N McCarthy, B Mearns, C Mearns, K Morgan-Jones, G Mortem, G Morton, B Moya, G Murphy, S Mutton, A Myers, T Nasser, J Navaratnam, S Nazir, S Nepal, K Nimako, L Nimako, O'Connor, A Patel, K Patel, V Phongsathorn, PA Pillai, M Poole, N Qureshi, S Ranjan, A Rehman, T Royal, T Samuels, E Scott, G Sekadde, A Sharma, G Sharp, S Shotton, O Simmons, P Singh, S Smith, K Sri Paranthamen, S Suresh, K Thevarajah, L Thomas, H Timms, N Tomasova, S Tucker, S Vara, C Vaz, S Weller, J White, M Wilde, I Wilkinson, C Williams, M Win, D Woosey, D Wright.

University Hospitals Of Morecambe Bay NHS Foundation Trust S Bari (PI), A Higham (Co-PI), M Al-Jibury, K Allison, F Andra, V Anu, C Bartlett, S Bhuiyan, L Bishop, K

Burns, A Davies, A Fielding, M Gorst, C Hay, J Keating, T Khan, F Mahmood, P Mallinder, S Peters, D Power, J Ritchie, K Simpson,
C Stokes, H Thatcher, A Varghese, T Wan, F Wood.

University Hospitals Of Derby and Burton NHS Foundation Trust T Bewick (PI), P Daniel (Co-PI), U Nanda (Co-PI), G Bell, C Downes, K English, A Fletcher, J Hampson, M Hayman, S Ohja, J Radford, K Riches, G Robinson, A Sathyanarayanan, F Scothern, L Wilcox, L Wright.

Portsmouth Hospitals NHS Trust T Brown (PI), J Andrews, M Baker-Moffatt, A Bamgboye, D Barnes, S Baryschpolec, L Bell, M Broadway, F Brogan, K Burrows, M Chauhan, A Chauhan, E Cowan, A Darbyshire, M David, H Downe, C Edwards, L Fox, A Gribbin, Y Harrington-Davies, E Hawes, A Hicks, E Hossain, S Howe, B Jones, B Longhurst, M Mamman, S McCready, C Minnis, M Moon, J Mouland, S Rose, H Rupani, K Scott, R Thornton, A Tiller, C Turner, M Wands, L Watkins, M White, L Wiffen, J Winter.

Bradford Teaching Hospitals NHS Foundation Trust D Saralaya (PI), N Akhtar, W Andrea, V Beckett, L Brear, V Drew, N Hawes, S Moss, S Oddie, K Regan, D Ryan-Wakeling, A Shenoy, K Storton, J Syson, R Wane.

University Hospitals Coventry and Warwickshire NHS Trust K Patel (PI), C Imray (Co-PI), N Aldridge, A Campbell, G Evans, E French, R Grenfell, S Hewins, D Hewitt, J Jones, R Kumar, E Mshengu, S Quenby, K Read, P Satodia, M Truslove.

Great Western Hospitals NHS Foundation Trust AL Kerry (PI), A Beale, A Brooks, C Browne, J Callaghan, B Chandrasekaran, C Coombs, R Davies, L Davies, T Elias, E Fowler, G Gowda, A Ipe, A Jaffery, Q Jones, L Kyeremeh, H Langton, C Lewis-Clarke, C Mackinlay, P Mappa, A Maxwell, W Mears, E Mousley, T Onyirioha, L Pannell, S Peglar, A Pereira, J Pointon, E Price, A Quayle, S Small, H Smith, E Stratton, M Tinkler, A Van Der Meer, E Wakefield, R Waller, M Walton, M Watters, L Whittam, T Wiliams, K Yein, V Zinyemba.

Calderdale and Huddersfield NHS Foundation Trust P Desai (PI), D Appleyard, S Dale, L Gledhill, J Goddard, J Greig, A Haigh, K Hanson, M Home, D Kelly, L Matapure, S Mellor, H Riley, M Robinson, K Sandhu, K Schwarz, L Shaw, L Terrett, M Usher, T Wood.

Medway NHS Foundation Trust R Sarkar (PI), I Ahmed, I Ahmed, S Ahmed, S-J Ambler, F Babatunde, S Banerjee, N Bhatia, L Brassington, F Brokke, D Bruce, B Cassimon, A Chengappa, N Divikar, C Donnelly, C Froneman, T Gower, H Harizaj, G Hettiarachchi, M Hollands, M Kamara, T Kyere-Diabour, K Lewiston, L Mires, A Mitchell, C Mizzi, K Naicker, I Petrou, MM Phulpoto, A Ross-Parker, I Ramadan, A Roy, A Ryan, E Samuels, T Sanctuary, R Sarkar, A Sharma, S Singham, J Sporrer, W Ul Hassan, P Vankayalapati, B Velan, L Vincent Smith, J Wood.

Mid Cheshire Hospitals NHS Foundation Trust D Maseda (PI), C Ball, K Best, G Bridgwood, C Brockelsby, T Brockley, J Brown, R Bujazia, S Clarke, C Dixon, S Dowson, C Emmett, H Farooq, D Fullerton, C Gabriel, S Hammersley, R Hum, T Jones, S Kay, M Kidd, D Lees, E Matovu, K McIntyre, M O'Brian, K Pagett, A Ritchings, S Smith, J Taylor, K Thomas.

East Suffolk and North Essex NHS Foundation Trust V Kushakovsky (PI), M Ramali (PI), S Alam, S Bartholomew, A Bataineh, D Beeby, S Bell, N Broughton, C Buckman, C Calver, J Campbell, C Chabo, M Chowdhury, K Cooke, C Driscoll, A Elden, H Eldew, N Entwistle, F Farnworth, R Francis, E Galloway, A Ghosh, G Gray, P Greenfield, M Hadjiandreou, H Hewer, MS Hossain, R Howard-Griffin, A Islam, E Jamieson, K Johannessen, SH Lee, R Lewis, R Lloyd, L Mabelin, D Morris, S Nallapareddy, HC Ooi, R Osagie, C Parkinson, H Prowse, B Purewal,P Ridley, V Rivers, J Rosier, S Sharma, A Sheik, R Smith, R Sreenivasan, A Taylor, P Tovey, K Turner, K Vithian, J Zhixin.

Sandwell and West Birmingham Hospitals NHS Trust S Clare (PI), MC Ahmed, Y Beuvink, K Blachford, S Clamp, J Colley, P De, B Gammon, A Hayes, L Henry, S Hussain, S Joseph, F Kinney, T Knight, R Kumar, W Leong, T Lim, B Mahay, Y Nupa, A Orme, S Potter, S Prew, N Rajaiah, A Rajasekaran, N Shah, S Sivakumar, L Smith, P Thozthumparambil, N Trudgill, A Turner, L Wagstaff, S Willetts, H Willis, M Yan.

The Royal Bournemouth and Christchurch Hospitals NHS Foundation Trust M Schuster Bruce (PI), D Baldwin, Z Clark, M Dale, D Griffiths, E Gunter, S Horler, T Joyce, M Keltos, S Kennard, N Lakeman, L Mallon, R Miln, S Orr, L Purandare, L Rogers, E Stride, L Vamplew.

Royal Berkshire NHS Foundation Trust M Frise (PI), R Arimoto, S Black, H Coles, H de Berker, A Fries, E Gabbitas, N Hasan, Z Htet, N Jacques, S Kashif, L Keating, S Leafe, Z Milne, B Mitchell, W Orchard, A Parmar, M Raffles, N Shields, M Thakker, M Thomas, H Wakefield, A Walden.

King's College Hospital NHS Foundation Trust M McPhail (PI), D Rao (Co-PI), J Adeyemi, J Aeron-Thomas, M Aissa, F Batalla, SM Candido, K Clark, K El Bouzidi, J Galloway, N Griffiths, A Gupta, Y Hu, E Jerome, N Kametas, N Long, H Martin, V Patel, T Pirani, S Ratcliff, S Rodrigues, B Sari, N Sikondari, J Smith, C Soto, A Te, R Uddin, M Waller, M West, C Williamson, M Yates, A Zamalloa.

Basildon and Thurrock University Hospitals NHS Foundation Trust K Thomas (PI), T George (Co-PI), Y Bernard, A Ikomi, L Jayasekera, L Kittridge, G Maloney, B Manoharan, M Mushabe, A Nicholson, A Pai, J Riches, J Samuel, N Setty, A Solesbury, D Southam, S Tisi, M Vertue, K Wadsworth.

York Teaching Hospital NHS Foundation Trust J Azam (PI), J Anderson, P Antill, S Appleby, P Armtrong, T Berriman, D Bull, O Clayton, A Corlett, D Crocombe, S Davies, K Elliott, L Fahel, J Ghosh, N Gott, D Greenwood, P Inns, K Mack, P Nikolaos,M O'Kane, P Ponnusamy, A Poole, R Proudfoot, S Roche, S Shahi, D Smith, B Sohail, R Thomas, A Turnbull, L Turner, J Wilson, D Yates.

University Hospitals Of North Midlands NHS Trust T Kemp (PI), W Al-Shamkhani, N Bandla, A Bland, N Bodasing, A Cadwgan, M Davies, L Diwakar, E Eaton, M Evans, A Farmer, F Farook, M Gellamucho, K Glover, I Hussain, L Korcierz, J Lee, J Machin, J Marshall, H McCreedy, G Muddegowda, Z Noori, H Parker, N Patel, A Quinn, M Ram, T Scott, C Thompson, J Tomlinson, H Turner, J Weeratunga, J White, P Wu.

North Cumbria Integrated Care NHS Foundation Trust C Graham (PI), A Abdelaziz, O Ali, G Bell, C Brewer, M Clapham, J Gregory, S Hanif, R Harper, M Lane, A McSkeane, U Poultney, K Poulton, S Pritchard, S Shah, C Smit, P Tzavaras, V Vasadi, T Wilson, A Wilson, D Zehnder.

Kettering General Hospital NHS Foundation Trust N Siddique (PI), K Adcock, S Adenwalla, A Ali, MZ Ashraf, MB Ashraq, S Ashton, H Asogan, H Aung, S Beyatli, S Coburn, D Ramdin, J Dales, R Deylami, A Elliott, S Gali, L Hollos, M Hussam El-Din, A Ibrahim, Y Jameel, RS Kusangaya, S Little, A Madu, G Margabanthu, D Ncomanzi, G Nikonovich, A Nisha James, Y Owoseni, D Patel, S Saunders, S Stapley, A Wazir, S White, J Wood.

NHS Greater Glasgow and Clyde: Glasgow Royal Infirmary K Puxty (PI), H Bayes (Co-I), J Alexander, L Bailey, A Begg, S Carmichael, S Cathcart, I Crawford, A Dougherty, K Gardiner, T Grandison, N Hickey, J Ireland, A Jamison, D Jenkins, J Johnstone, L Martin, M McIntyre, A-M Munro, S Nelson, H Peddie, G Piper, D Rimmer, G Semple, S Thornton.

London North West University Healthcare NHS Trust A Whittington (PI), M Adamus, J Barrett, F Cawa, S Chhabra, E Dhillon, J Goodall, R Gravell, A Gupta-Wright, S Gurram, S Isralls, M Lang, C Macleod, J Milburn, K Muralidhara, B Nayar, O Ojo, P Papineni, V Parris, M Patel, S Quaid, J Sethi, B Tyagi, LC Vaccari, G Wallis, E Watson.

Chelsea and Westminster Hospital NHS Foundation Trust P Shah (PI), B Mann (Co-PI), K Alizaedeh, M Al-Obaidi, A Barker, C Bautista, M Boffito, M Bourke, R Bull, C Caneja, J Carungcong, P Costa, E Dwyer, C Fernandez, J Girling, E Hamlyn, A Holyome, L Horsford, N Hynes, M Johnson, U Kirwan, C Lloyd, S Maheswaran, M Martineau, M Nelson, T Ngan, K Nundlall, T Peters, A Sayan, A Schoolmeesters, M-L Svensson, A Tana, M Thankachen, A Thayanandan, E Vainieri, C Winpenny, O Zibdeh.

North Bristol NHS Trust N Maskell (PI), J Dodd (Co-PI), S Barratt (Co-PI), H Adamali, P Creber, A Bibby, A Jeyabalan, E Moran, M Alvarez, D Arnold, G Hamilton, D Higbee, A Dipper, H Welch, S Bevins, R Bhatnagar, C Burden, A Cashell, H Cheshire, S Clarke, A Clive, K Farmer, L Jennings, H Lee, H McNally, A Milne, A Morley, E Perry, V Sandrey, K Smith, L Solomon, L Stadon, R Wach, C Watkins.

Aneurin Bevan University LHB T Szakmany (PI), S Champanerkar (Co-I), A Griffiths (Co-I), AA Ionescu (Co-I), C Somashekar (Co-I), K Zalewska (Co-I), L Aitken, E Baker, D Barnett, M Brouns, S Cherian, R Codd, S Cutler, W Davies, A Dell, M Edwards, S Edwards, N Hawkins, S Hodge, R Hughes, C Ivenso, T James, M Jones, S Jones, J MacCormac, G Marshall, S McKain, H Nassa, J Northfield, S Palmer, L Peter, C Price, M Pynn, A Roynon Reed, L Shipp, J Singh, M Singh, K Swarnkar, P Torabi, E Wall, A Waters, K Wild, M Winstanley, K Wyness.

East Kent Hospitals University NHS Foundation Trust N Richardson (PI), A Alegria (PI), R Kapoor (PI), C Linares (Co-PI), K Adegoke, A Alegria, L Allen, G Boehmer, N Crisp, J Deery, J Hansen, C Hargreaves, R Hulbert, A Knight, D Loader, J McAndrew, M Montasser, A Moon, C Oboh, P Offord, C Price, A Rajasri, J Rand, D Stephensen, S Stirrup, S Tilbey, S Turney, V Vasu, M Venditti, H Weston, Z Woodward.

Southend University Hospital NHS Foundation Trust G Koduri (PI), S Gokaraju (Co-PI), F Hayes (Co-PI), VW Vijayaraghavan Nalini (Co-PI), V Gupta, P Harman.

Sherwood Forest Hospitals NHS Foundation Trust M Roberts (PI), K Amsha (Co-I), G Cox (Co-I), N Downer (Co-I), D Hodgson (Co-I), J Hutchinson (Co-I), S Kalsoom (Co-I), A Molyneux (Co-I), Z Noor (Co-I), L Allsop, P Buckley, L Dunn, M Gill, C Heeley, M Holmes, C Moulds, D Nash, J Rajeswary, S Shelton, S Smith, I Wynter, M Yanney.

County Durham and Darlington NHS Foundation Trust J Limb (PI), V Atkinson, M Birt, A Cowton, V Craig, D Egginton, A Ivy, D Jayachandran, J Jennings, A Kay, S Mcauliffe, S Naylor, G Nyamugunduru, K Potts, P Ranka, G Rogers, S Sen, J Temple, S Wadd, H Walters, J Yorke.

Imperial College Healthcare NHS Trust G Cooke (PI), Z Al-Saadi, A Daunt, L Evison, C Gale, M Gibani, B Jones, J Labao, N Madeja, H McLachlan, A Perry, R Thomas, E Whittaker, C Wignall, P Wilding, L Young, C Yu.

The Dudley Group NHS Foundation Trust H Ashby (PI), P Amy, S Ashman-Flavell, S de Silva, J Dean, N Fisher, E Forsey, J Frost,S Jenkins, D Kaur, A Lubina Solomon, S Mahadevan-Bava, T Mahendiran, M O'Toole, S Pinches, D Rattehalli, U Sinha, M Subramanian, J Vamvakopoulos, S Waidyanatha.

Sheffield Teaching Hospitals NHS Foundation Trust P Collini (PI), A Ang, J Belcher, KW Chin, D Cohen, J Cole, R Condliffe, M Cribb, S Curran, T Darton, D de Fonseka, T de Silva, E Ferriman, J Greig, MU Haq, E Headon, C Holden, L Hunt, E Hurditch, T Kitching, L Lewis,

T Locke, L Mair, P May, J Meiring, J Middle, P Morris, L Passby, R Payne, S Renshaw, D Sammut, S Sherwin, M Sterrenburg,
B Stone, M Surtees, A Telfer, R Thompson, N Vethanayagam, R West.

Hampshire Hospitals NHS Foundation Trust R Partridge (PI), A Goldsmith (Co-PI), Y Abed El Khaleq, A-M Arias, E Bevan, J-A Conyngham, E Cox, E Defever, A Heath, B King, E Levell, X Liu, A Nejad, F Stourton, R Thomas, D Trodd, B White, G Whitlingum, L Winckworth, C Wrey Brown, S Zagalo.

Barking, Havering and Redbridge University Hospitals NHS Trust M Phull (PI), A Misbahuddin (Co-PI), A Umaipalan (Co-PI), J Ah-Chuen, A Ainsley, G Baijiu, A Basumatary, C Calderwood, P Dugh, K Dunne, P Greaves, K Hunt, M Islam, V Katsande, MR Khan, S King, A Mohamed, N Nagesh, D Nicholls, N O'Brien, L Parker, T Pogreban, L Rosaro, E Salciute, M Sharma, H Smith, E Visentin, L Walshaw.

NHS Greater Glasgow and Clyde: Royal Alexandra Hospital A Corfield (PI), C Clark, P Clark, S Drysdale, M Heydtmann, E Hughes, L Imam-Gutierrez, I Keith, D McGlynn, G Ray, N Rodden, K Rooney, N Thomson.

St George's University Hospitals NHS Foundation Trust T Bicanic (PI), T Harrison (Co-PI), A Adebiyi, S Drysdale, A Khalil, T Samakomva, St Georges COVID 19 Research Team.

James Paget University Hospitals NHS Foundation Trust J Patrick (PI), B Burton (Co-I), J Chapman, V Choudhary, C Hacon, K Mackintosh, H Sutherland, M Whelband, E Wilhelmsen.

West Suffolk NHS Foundation Trust M Moody (PI), S Barkha (Sub-PI), S Bhagat, H Cockerill, J Godden, J Kellett, T Murray, P Oats, A Saraswatula, A Williams, L Wood.

Leeds Teaching Hospitals NHS Trust J Minton (PI), S Ahmed, A Ashworth, J Bailey, M Baum, L Bonney, J Calderwood, C Coupland, M Crow, J Furness, K Johnson, A Jones, M Kacar, P Lewthwaite, G Lockley-Ault, F McGill, J Murira, Z Mustufvi, S O'Riordan, K Robinson, R Saman, J Stahle, S Straw, A Westwood, N Window.

The Rotherham NHS Foundation Trust A Hormis (PI), C Brown, D Collier, C Dixon, J Field, R Walker, L Zeidan.

Blackpool Teaching Hospitals NHS Foundation Trust J Cupitt (PI), N Ahmed, O Assaf, A Barnett, L Benham, P Bradley, Z Bradshaw, T Capstick, R Downes, J Navin, N Latt, R McDonald, A Mulla, E Mutema, A Parker, S Preston, N Slawson, V Vasudevan, J Wilson, A Zmierczak.

South Tyneside and Sunderland NHS Foundation Trust E Fuller (PI), A MacNair (Co-PI), M Rangar (Co-PI), N Mullen PI (Paediatrics), C Brown, A Burns, C Caroline, R Davidson, M Dickson, B Duncan, N Elkaram, I Emmerson, L Fairlie, M Hashimm, J Henderson, K Hinshaw, J Holden, S Laybourne, P Madgwick, K Martin, M McKee, J Moore, P Murphy, R Shahrul, A Smith, L Smith, M Smith, B Stidolph, L Terry, A Trotter, F Wakinshaw, M Walton.

University Hospitals Bristol and Weston NHS Foundation Trust N Blencowe (PI), P Singhal (Co-PI), M Abraham, B Al-Ramadhani, A Archer, G Aziz, A Balcombe, K Bateman, M Baxter, N Bell, M Beresford, J Bernatoniene, D Bhojwani, S Biggs, J Blazeby, K Bobruk, N Brown, L Buckley, P Butler, C Caws, E Chakkarapani, B Chivima, C Clemente, K Cobain, D Cotterill, E Courtney, S Cowman, K Coy, K Curtis, P Davis, O Drewett, H Dymond, K Edgerley, M Ekoi, B Evans, T Farmery, N Fineman, A Finn, L Gamble, F Garty, B Gibbison, L Gourbault, D Grant, K Gregory, M Griffin, R Groome, M Hamdollah-Zadeh, A Hannington, A Hindmarsh, N Holling, R Houilhan, J Hrycaiczuk, H Hudson, K Hurley, R Jarvis, B Jeffs, A Jones, RE

Jones, E Kirkham, R Kumar, M Kurdy, S Lang, L Leandro, H Legge,F Loro, A Low, H Martin, L McCullagh, G McMahon, L Millett, K Millington, J Mok, J-H Moon,L Morgan, S Mulligan, C O'Donovan, E Payne, C Penman, C Plumptre, A Ramanan, J Ramirez, S Ratcliffe, J Robinson, M Roderick, S Scattergood, A Schadenberg, R Sheppeard, C Shioi, D Simpson, A Skorko,R Squires, M Stuttard, P Sugden, S Sundar, T Swart, E Swift, K Thompson, S Turner, K Turner, A Tyer, S Vergnano, R Vincent, J Williams, J Willis, H Winter, L Woollen, R Wright, A Younes Ibrahim.

Lancashire Teaching Hospitals NHS Foundation Trust S Laha (PI), A Ashfaq, A Bellis, M Chiu, W Choon Kon Yune, M Deeley, W Flesher, K Gandhi, S Gudur, R Gupta, A Huckle, G Long, A McCarrick, J Mills, S Nawaz, O Parikh, D Rengan, S Sherridan, B So, R Sonia, K Spinks, A-M Timoroksa, B Vernon, A Williams, K Williams, H Wu.

Wirral University Teaching Hospital NHS Foundation Trust A Wight (PI), L Bailey, S Brownlee, A Hufton, R Jacob, H Kerss, J McEntee, R Myagerimath, M Parsonage, H Peake, D Pearson, S Rath, R Saunders, B Spencer, A Suliman, S Sutton, H Tan, D Tarpey, L Thompson, T Thornton, Z Wahbi.

United Lincolnshire Hospitals NHS Trust M Chablani (PI), R Barber (Co-PI), S Archer, R Barber, S Butler, A Chingale, C Flood, O Francis, A Kirkby, R Mishra, K Netherton, L Osborne, A Reddy, A Sloan,

Walsall Healthcare NHS Trust A Garg (PI), S al-Hity, B Allman, L Botfield, A Bowes, L Boyd, W Campbell, Z Chandler, J Collins, GK Dhaliwal, L Dwarakanath, A Farg, A Foot, A Gondal, T Gupta, T Jemima, O Khan, J Khatri, R Krishnamurthy, H Mahmoud, R Marsh, R Mason, M Matonhodze, S Misra, A Naqvi, E Nicholls, SA Nortcliffe, SW Odelberg, M Phipps, A Plant, K Punia, H Qureshi, P Ranga, A Raymond-White, N Richardson, L Rogers, A Sheikh, A Srirajamadhuveeti, N Sunni, R Turel, E Virgilio, F Wyn-Griffiths.

Royal Papworth Hospital NHS Foundation Trust RC Rintoul (PI), F Bottrill, A Butchart, K Dorey, R Druyeh, M Earwaker, C East, S Fielding, A Fofana, C Galloway, L Garner, A Gladwell, V Hughes, R Hussey, N Jones, S Mepham, A Michael, H Munday, K Paques, H Parfrey, C Pathvardhan, G Polwarth, A Rubino, S Webb, S Woods, K Woodall, A Vuylsteke, J Zamikula.

The Newcastle Upon Tyne Hospitals NHS Foundation Trust A De Soyza (PI), R Agbeko, K Baker, E Cameron, Q Campbell Hewson, C Duncan, M Emonts, A Fenn, A Greenhalgh, A Hanrath, K Houghton, D Jerry, G Jones, S Kelly, N Lane, P McAlinden, O Mohammed, R Obukofe, J Parker, A Patience, B Payne, D Price, S Robson, E Stephenson, R Welton, S West, E Wong, F Yelnoorkar.

Ashford and St Peter's Hospitals NHS Foundation Trust C Russo (PI), M Croft, V Frost, M Gavrila, K Gibson, A Glennon, C Gray, N Holland, J Law, R Pereira, P Reynolds, H Tarft, J Thomas, L Walding, A Williams.

Kingston Hospital NHS Foundation Trust S Mahendran (PI), A Joseph (Co-PI), A Baggaley, G Bambridge, F Bazari, P Beak, A Conroy, J Crooks, B Crowe, E Donnelly, A Edwards, S El-Sayeh, A Feben, J Fox, R Gisby, J Haugh, R Herdman-Grant, D Jajbhay, O James, S Jones, A Joseph, T Leahy, M Madden, J May, G McKnight, L Mumelj, G Natarajan, J Poxon, A Ratnakumar, T Sanderson, R Simms, D Sivakumaran, A Sothinathan, A Swain, S Swinhoe, M Taylor, M Trowsdale Stannard, J Vance-Daniel, H Warren-Miell, T Woodhead, D Zheng.

Liverpool University Hospitals NHS Foundation Trust S Todd (PI), I Welters (Co-PI), D Wootton (Co-PI), A Al Balushi, D Barr, J Byrne, D Coey, T Cross, J Cruise, K Haigh, C Hall, M Harrison, S Hope, K Hunter, S Iyer, A Jackson, J Keogan, J Lewis, P Lopez, C Lowe, M

Middleton, M Nugent, L Pauls, I Quayle, S Raghunath, M Riley, J Sedano, D Shaw, S Stevenson, A Stockdale, R Tangney, V Waugh, K Williams.

Bedford Hospital NHS Trust E Thomas (PI), D Bagmane (Co-PI), B Jallow (Co-PI), I Nadeem (Co-PI), M Negmeldin (Co-PI), A Vaidya (Co-PI), A Amjad, A Anthony-Pillai, I Armata, R Arora, R Bhanot, D Callum, P Chrysostomou, F De Santana Miranda, S Farnworth, N Fatimah, L Grosu, A Haddad, M Hikmat, UF Khatana, I Koopmans, E Lister, R Lorusso, N Nathaniel, CS Ong, K Pandya, M Penacerrada, S Rahama, L Salih, W Tan, S Trussell, J Valentine, R Wulandari.

Whittington Health NHS Trust C Parmar (PI), WW Ang, M Christy, P Dlouhy, K Gilbert, F Green, M Kousteni, J Flor, S Myers, L Ma, S Rudrakumar, J Sabale, K Simpson, L Veys, N Wolff, A Zuriaga-Alvaro.

Bolton NHS Foundation Trust M Balasubramaniam (PI), C Subudhi (Co-PI), A Ajmi, R Ahmed, A Al-Asadi, A Amin, M Bhalme, Z Carrington, J Chadwick, S Cocks, C Dawe, A Eusuf, S Farzana, P Hill, R Holmes, G Hughes, R Hull, K Ibrahim,M Ijaz, S Khurana, S Latham, K Lipscomb, JP Lomas, N Natarajan, D Nethercott, D Obeng, V Priyash, K Rhead, M Saleh, Z Shehata, R Sime, S Singh, R Smith, E Tanton, D Tewkesbury, S Thornton, N Wang, M Watts, I Webster.

NHS Greater Glasgow and Clyde: Queen Elizabeth University Hospital M Sim (Co-PI), KG Blyth (Co-PI), L Jawaheer (Co-PI), L Pollock (Co-PI), S Wishart (Co-PI), M McGettrick (Sub-I), J Rollo (Sub-I), N Baxter, J Ferguson, K Ferguson, S Henderson, S Kennedy-Hay, A Kidd, MA Ledingham, M Lowe, R McDougall, M McFadden, N McGlinchey, L McKay, B McLaren, C McParland, J McTaggart, J Millar, L Rooney, H Stubbs, M Wilson.

Western Sussex Hospitals NHS Foundation Trust L Hodgson (PI), M Margarson (Co-PI), L Albon, M Bailey, Y Baird, I Balagosa, A Brereton, S Bullard, A Butler, V Cannons, P Carr, C Chandler, G Chow, V Dandavate, R Duckitt, A Elkhawad, S Floyd, L Folkes, K Forcer, H Fox, S Funnell, N Gent, A Ghazanfar, J Gilbert, R Gomez-Marcos, N Hedger, K Hedges, D Helm, A Hetreed, G Hobden, H Htet, D Hunt, P Jane, D Jennings, A Kanish, R Khan, S Kimber, K King, Z Krejcarova, T Leckie, M Linney, L Lipskis, C Long, J Margalef, T Martindale, A Matthew, M McCarthy, P McGlone, E Meadows, S Moore, T Moore, S Murphy, M Nelmes, L Nguyen, R Njafuh, L Norman, N Numbere, M Parson, E Pineles, M Purcell, L Ramsawak, C Ranns, D Raynard, D Reynish, L Riddles, C Ridley, E Robinson, J Russell, T Shafi, S Sinha, S Stone, P Tate, B Thillainathan, Y Thirlwall, R Venn, J Villiers, N White, J Wileman, E Yates.

Brighton and Sussex University Hospitals NHS Trust M Llewelyn (PI), H Brown, E Barbon, G Bassett, L Bennett, A Bexley, Z Cipinova, J Gaylard, Z He, C Laycock, D Mullan, C Richardson, V Sellick, D Skinner, M Smith.

NHS Lothian: Western General Hospital O Koch (PI), A Abu-Arafeh, E Allen, C Balmforth, A Barnett-Vanes, R Baruah, S Blackley, S Clifford, A Clarke, M Curtin, M Evans, C Ferguson, S Ferguson, N Fethers, N Freeman, E Godden, R Harrison, B Hastings, S Htwe, AJW Kwek, O Lloyd, C Mackintosh, A MacRaild, W Mahmood, E Mahony, J McCrae, E Moatt, S Morris, C Mutch, K Nunn, M Perry, J Rhodes, N Rodgers, A Shepherd, R Sutherland, A Tasiou, A Tufail, D Waters, T Wilkinson, R Woodfield, J Wubetu.

Southern HSC Trust R Convery (PI), J Brannigan, D Cosgrove, C McCullough, D McFarland, R McNulty, S Sands, O Thompson.

Northern Lincolnshire and Goole NHS Foundation Trust A Mitra (PI), MN Akhtar, H Al-Moasseb, S Amamou, T Behan, S Biuk, M Brazil, M Brocken, C Burnett, C Chatha, M Cheeseman, L-J Cottam, T Cruz Cervera, K Dent, C Dyball, K Edwards, R Elmahdi, Q Farah,

S Farooq, S Gooseman, J Hargreaves, MA Haroon, J Hatton, E Heeney, J Hill, E Horsley, R Hossain, D Hutchinson, J Hyde-Wyatt, M Iqbal, N James, S Khalil, M Madhusudhana, A Marriott, MT Masood, K Mellows, R Miller, U Nasir, M Newton, GCE Ngui, S Pearson, C Pendlebury, R Pollard, N Pothina, D Potoczna, SD Raha, A Rehan, SAS Rizvi, A Saffy, K Shams, C Shaw, A Shirgaonkar, S Spencer, R Stead, R Sundhar, D Taylor, E Thein, L Warnock, KY Wong.

Norfolk and Norwich University Hospitals NHS Foundation Trust E Mishra (PI) C Atkins (Co-PI), KS Myint (Co-PI), J Nortje (Co-PI), D Archer, M Cambell-Kelly, P Clarke, L Coke, M Cornwell, H Gorrick, A Haestier, M Harmer, L Harris, L Hudig, L Jones, J Keshet-Price, E Kolokouri, V Licence, E Malone, M-A Morris, G Randell.

Shrewsbury and Telford Hospital NHS Trust J Moon (PI), N Biswas, A Bowes, H Button, M Carnhan, S Deshpande, C Fenton, M Ibrahim, J Jones, H Millward, M Rees, N Schunke, J Stickley, M Tadros, H Tivenan.

Swansea Bay University Local Health Board B Healy (PI), S Bareford, I Blyth, A Bone, E Brinkworth, R Chudleigh, Y Ellis, S Georges, S Green, R Harford, J Harris, A Holborow, C Johnston, P Jones, M Krishnan, N Leopold, F Morris, A Mughal, E Pratt, T Rees, G Saleeb, J Watts, M Williams.

St Helens and Knowsley Teaching Hospitals NHS Trust G Barton (PI), S Dealing, R Garr, S Greer, N Hornby, S Mayor, A McCairn, S Rao.

Royal Devon and Exeter NHS Foundation Trust M Masoli (PI), H Bakere, A Bowring, P Czylok, L Dobson, A Forrest, E Goodwin, H Gower, L Knowles, A Mackey, V Mariano, L Mckie, P Mitchelmore, L Morgan, R Oram, N Osborne, I Seaton, R Sheridan, J Tipper, S Wilkins, N Withers.

Dorset County Hospital NHS Foundation Trust J Chambers (PI), J Birch, L Bough, J Graves, S Horton, R Thomas, W Verling, S Williams, P Williams, B Winter-Goodwin, S Wiseman, D Wixted.

The Queen Elizabeth Hospital, King's Lynn, NHS Foundation Trust M Blunt (PI), J Ali, K Beaumont, K Bishop, H Bloxham, P Chan, Z Coton, H Curgenven, M Elsaadany, T Fuller, M Iqbal, M Israa, S Jeddi, SA Kamerkar, EET Lim, E Nadar, K Naguleswaran, O Poluyi, G Rewitzky, S Ruff, A Velusamy.

Gloucestershire Hospitals NHS Foundation Trust C Sharp (PI), F Ahmed, O Barker, O Bintcliffe, P Brown, R Bulbulia, J Collinson, T Cope, A Creamer, C Davies, W Doherty, M Fredlund, J Glass, S Harrington, A Hill, H Iftikhar, M James, C Lim, S Message, J Ord, T Pickett, A Simpson, M Slade, H Uru, D Ward, R Woolf.

NHS Tayside: Ninewells Hospital J Chalmers (PI), H Abo-Leyah, C Almadenboyle, C Deas, H Loftus, A Nicoll, L Smith, A Strachan, J Taylor, C Tee.

Royal Free London NHS Foundation Trust B Caplin (PI), H Tahir (Co-PI), R Abdul-Kadir, M Anderson, GR Badhan, E Cheung, V Conteh, R Davies, H Hughes, V Jennings, H Mahdi, P Patel, T Sobande.

NHS Grampian: Aberdeen Royal Infirmary J Cooper (PI), V Bateman, M Black, R Brittain-Long, K Colville, D Counter, S Devkota, P Dospinescu, J Irvine, C Kaye, A Khan, R Laing, MJ MacLeod, J McLay, D Miller, K Norris, R Soiza, V Taylor.

Croydon Health Services NHS Trust T Castiello (PI), J Adabie-Ankrah, G Adkins, B Ajay, S Ashok, A Dean, S Dillane, V Florence, D Griffiths, I Griffiths, C Jones, A Latheef, S Lee, J McCammon, S Patel, A Raghunathan, J Talbot-Ponsonby, G Upson, G Upson.

The Royal Wolverhampton NHS Trust S Gopal (PI), R Barlow, CH Cheong, D Churchill, K Davies, M Green, N Harris, A Kumar, S Metherell, S Milgate, L Radford, J Rogers, A Smallwood.

Southport and Ormskirk Hospital NHS Trust S Pintus (PI), A Ahmed (Co-I), A Nune (Co-I), S Abdelbadiee, L Afari, L Aitchson, A Ali, S Asam, N Babajan, B Bainton, L Bishop, K Choudhary, A Christie, R Cox, M Diwan, W Gaba, H Gibson, Z Haslam, A Hassan, C Hutchcroft, M Jackson, A Liaretidou, M Mahmood, E McDonald, A Morris, M Morrison, N Ndoumbe, S O'Brien, S Rehman, N Shami, L Smith, L Undrell, K Wahdati, M Wood.

Mid Yorkshire Hospitals NHS Trust A Rose (PI), J Ashcroft (Co-I), P Blaxill (Co-I), S Bond (Co-I), A Dwarakanath (Co-I), C Hettiarachchi (Co-I), B Sloan (Co-I), S Taylor (Co-I), M Thirumaran (Co-I), R Beckitt, S Buckley, G Castle, E Clayton, N De Vere, J Ellam, D Gomersall, S Gordon, C Hutsby, R Kousar, K Lindley, S Oddy, L Slater, B Taylor.

Tameside and Glossop Integrated Care NHS Foundation Trust B Ryan (PI), A Abraheem, C Afnan, B Ahmed, O Ahmed, M Anim-Somuah, A Armitage, P Arora, M Beecroft, A-T Butt, J Fallon, J Foster, I Foulds, N Garlick, H Ghanayem, S Gulati, R Hafiz-Ur-Rehman, M Hamie, A Hewetson, B Ho, B Horsham, W Hughes, W Hulse, A Humphries, M Hussain, N Johal, E Jude, M Kelly, A Kendall-Smith, M Khan, R Law, J Majumdar, J McCormick, O Mercer, T Mirza, B Obale, P Potla, S Pudi, K Qureshi, M Rafique, R Rana, R Roberts, J Roddy, C Rolls, M Sammut, H Savill, M Saxton, V Turner, A Tyzack.

Doncaster and Bassetlaw Teaching Hospitals NHS Foundation Trust C-H Wong (PI), A Adeni, J Allen, S Allen, A Bassaly, M Beaumont, P Cawley, R Chadwick, R Codling, F Dunning, A Ermenyi, D Grabowska, D Graham, N Hammoud, G Herdman, M Highcock, S Hussain, N Khota, G Kirkman, C Knapp, M Kyi, A Mandal, J Maskill, V Maxwell, S McGonagle, S Mukhtar, A Nasimudeen, A Natarajan, D Pryor, D Sagar, N Saqib, P Shannon, Y Syed, D Trushell-Pottinger, L Warren, N Wilkinson, T Wilson.

Mid Essex Hospital Services NHS Trust A Hughes (PI), J Radhakrishnan (Co-PI), T Camburn, C Catley, E Dawson, C Fox, N Fox, H Gerrish, S Gibson, H Guth, F McNeela, A Rao, S Reid, B Singizi, S Smolen, S Williams, L Willsher, J Wootton.

The Princess Alexandra Hospital NHS Trust U Ekeowa (PI), Q Shah (Co-PI), M Anwar, G Arunachalam, B Badal, K Bamunuarachchi, G Cook, A Daniel, J Finn, C Freer, A Gani, E Haworth, E Holmes, L Hughes, K Ixer, G Lucas, C Muir, S Naik, R Ragatha, P Russell, R Saha, L Sandhu, E Shpuza, N Staines, S Waring, L Wee, F Weidi, T White.

Maidstone and Tunbridge Wells NHS Trust K Cox (PI), A Abbott, S Anandappa, B Babiker, C Bailey, M Barbosa, G Chamberlain, D Datta, M Davey, R Gowda, R Hammond-Hall, E Harlock, C Hart, A Henderson, SY Husaini, E Hutchinson, T-K Loke, S Matthew, R Nemane, I Pamphlett, C Pegg, A Richards, S Siddavaram, H Slater, G Sluga, O Solademi, P Tsang.

Cambridge University Hospitals NHS Foundation Trust M Knolle (PI), E Gkrania-Klotsas (Co-PI), P Bailey, K Beardsal, R Bousefield, K Bunclark, S Burge, J Chung, T Dymond, A Edwards, M Fisk, K Gajewska-Knapik, J Galloway, C Harris, A Jha, R Kumar, K Leonard, C Ma, A Martinelli, Z McIntyre, N Pathan, S Rossi, J Sahota, G Stewart, A Sutton-Cole, E Torok, M Toshner, C Yong.

East Lancashire Hospitals NHS Trust S Chukkambotla (PI), S Duberley, W Goddard, K Marsden.

Milton Keynes University Hospital NHS Foundation Trust R Stewart (PI), S Bowman (Co-PI), A Chakraborty (Co-PI), L How (Co-PI), D Mital (Co-PI), L Anguvaa, J Bae, G Bega, S Bosompem, E Clare, A Dooley, S Fox, J Mead, S Mehdi, L Mew, L Moran, E Mwaura, M Nathvani, A Oakley, A Rose, A Sanaullah, D Scaletta, S Shah, L Siamia, J Smith, O Spring, S Velankar, F Williams, L Wren, F Wright.

Lewisham and Greenwich NHS Trust S Kegg (PI), A Aghababaie, H Azzoug, E Bates, M Chakravorty, K Chan, F Chukwunonyerem, E Gardiner, A Hastings, D Jegede, J Juhl, S Khatun, M Magriplis, C Milliken, J Muglu, D Mukimbiri, M Nadheem, T Nair, M Nyirenda, T Oconnor, T Ogbara, R Olaiya, C Onyeagor, V Palaniappan, A Pieris, S Pilgrim, C Saad, N Sengreen, A Taylor, K Wesseldine, M Woodman.

Warrington and Halton Teaching Hospitals NHS Foundation Trust M Murthy (PI), R Arya, R Chan, L Connell, L Ditchfield, N Marriott, H Prady, L Roughley, H Whittle.

South Eastern HSC Trust D Alderdice (PI), J Courtney (Co-I), J Elder (Co-I), D Hart (Co-I), K Henry (Co-I), R Hewitt (Co-I), A Kerr (Co-I), J McKeever (Co-I), C O'Gorman (Co-I), S Rowan (Co-I), T Trinick (Co-I), B Valecka (Co-I), P Yew (Co-I), V Adell, J Baker, A Campbell, J Foreman, P Gillen, S Graham, S Hagan, L Hammond, J MacIntyre, A Smith, G Young.

NHS Fife D Dhasmana (PI), F Adam, K Aniruddhan, J Boyd, N Bulteel, P Cochrane, K Gray, L Hogg, S Iwanikiw,M Macmahon, A Morrow, J Penman, H Sheridan, D Sloan, C Stewart.

Royal Cornwall Hospitals NHS Trust D Browne (PI), H Chenoweth, F Hammonds, L Jones, E Laity, R Sargent, K Watkins, L Welch.

George Eliot Hospital NHS Trust S George (PI), K Ellis, V Gulia, J Gunn, E Hoverd, T Kannan, R Musanhu, N Navaneetham, D Suter.

NHS Lanarkshire: University Hospital Monklands M Patel (PI), C McGoldrick (Co-PI), C Beith, L Ferguson, L Glass, P Grant, S MacFadyen, A McAlpine, M McLaughlin, S Rundell, C Sykes, M Taylor, B Welsh.

Stockport NHS Foundation Trust R Stanciu (PI), M Afridi, S Bennett, L Brown, C Cooper, A Davison, D Eleanor, J Farthing, A Ferrera, P Haywood, C Heal, H Jackson, J Johnston, A Lloyd, R Owen, A Pemberton, F Rahim, H Robinson, N Sadiq, R Samlal, V Subramanian, D Suresh, H Wieringa, I Wright.

NHS Lanarkshire: University Hospital Wishaw M Patel (PI), K Black, R Boyle, S Clements, J Fleming, L Glass, L Hamilton, E Jarvie, C MacDonald, D Vigni, B Welsh, P Wu.

Poole Hospital NHS Foundation Trust H Reschreiter (PI), S August, C Barclay, S Blunden, S Bokhandi, J Camsooksai, S Chessell, C Colvin, J Dube, S Grigsby, C Humphrey, S Jenkins, S Patch, A Shah, M Tighe, L Vinayakarao, B Wadams, E Woodward, M Woolcock.

Gateshead Health NHS Foundation Trust R Allcock (PI), M Armstrong, J Barbour, A Dale, V Deshpande, I Hashmi, E Johns, D Mansour, B McClelland, C McDonald, C Moller-Christensen, R Petch, R Sharma, L Southern, G Stiller.

NHS Forth Valley: Forth Valley Royal Hospital M Spears (PI), A Baggott, G Clark, J Donnachie, S Huda, G Jayasekera, I Macpherson, M Maycock, J McMinn, A Pearson, L Prentice, C Rafique, D Salutous, M Stewart, L Symon, A Todd, P Turner.

Royal United Hospitals Bath NHS Foundation Trust J Suntharalingam (PI), J Avis, S Burnard, J Fiquet, J Ford, O Griffiths, R Hamlin, S Jones, J Macaro, R MacKenzie Ross, C Marchand, S Mitchard, A Palmer, L Ramos, M Rich, J Rossdale, S Sturney, J Tyler.

University Hospital Southampton NHS Foundation Trust S Fletcher (PI), K Cathie, S Chabane, M Coleman, SN Faust, CE Jones, T Jones, S Michael, M Petrova, L Presland, A Procter, T Sass, M Shaji, C Silva Moniz, T Thomas, S Triggs, C Watkins, S Wellstead, H Wheeler.

University Hospitals Plymouth NHS Trust D Lewis (PI), D Affleck, O Anichtchik, K Bennett, M Cramp, J Day, M Dobranszky Oroian, E Freeman, C Morton, H Notman, C Orr, A Patrick, L Pritchard, J Shawe, H Tan.

Wye Valley NHS Trust I DuRand (PI), P Ryan (Deputy PI), J Al-Fori, J Birch, N Bray, A Carrasco, M Cohn, E Collins, S Cooper, A Davies, M Evans, K Hammerton, S Meyrick, B Mwale, L Myslivecek, C Seagrave, F Suliman, S Turner, J Woolley.

Worcestershire Acute Hospitals NHS Trust C Hooper (PI), K Austin, T Dawson, A Durie, C Hillman-Cooper, M Ling, J Tyler, P Watson, H Wood.

Hull University Teaching Hospitals NHS Trust N Easom (PI), K Adams, L Baldwin, G Barlow, R Barton, H Bexhell, A James, X Kassianides, M Kolodziej, P Lillie, V Mathew, S Mongolu, IA Muazzam, P O'Reilly, C Philbey, B Pickwell-Smith, L Rollins, T Sathyapalan, K Sivakumar, H Yates.

Royal Surrey County Hospital NHS Foundation Trust K McCullough (PI), C Beazley, H Blackman, P Carvelli, P Chaturvedi, B Creagh-Brown, J De Vos, S Donlon, C Everden, J Fisher, E Gallagher, D Greene, O Hanci, E Harrod, N Jeffreys, J Jones, R Jordache, N Michalak, O Mohamed, S Mtuwa, K Penhaligon, V Pristopan, M Sanju, E Smith, S Stone, S Tluk.

Cwm Taf Morgannwg University LHB C Lynch (PI), B Deacon, S Eccles, B Gibson, C Lai, L Margarit, DS Nair, S Owen, L Roche, S Sathe.

Betsi Cadwaladr LHB: Glan Clwyd Hospital D Menzies (PI), A Abou-Haggar, S Ambalavanan, K Darlington, F Davies, G Davis, I Davis, J Easton, T Grenier, S Horrocks, R Lean, J Lewis, R Poyner, R Pugh, X Qui, S Rees, N Sengupta, H Williams.

University College London Hospitals NHS Foundation Trust H Esmail (PI), RS Heyderman (Co-PI), DAJ Moore (Co-PI), F Beynon, PN Bodalia, XHS Chan, CY Chung, D Crilly, J Gahir, L Germain, J Glanville, E Kilich, N Lack, N Platt, I Skorupinska, M Skorupinska, J Spillane, N Z Fard.

East and North Hertfordshire NHS Trust M Chaudhury (PI), C Cruz (Co-I), M Ebon (Co-I), N Pattison (Co-I), J Asplin, P Baker, D Banner, H Beadle, C Cruz, S Dabbagh, M Ebon, V Elliott, P Ferranti, J Gilmore, S Gohil, A Hood, T Ingle, E Jenner, Z Kantor, J Mathers, K Mccord, K Narula, J Newman, Y Odedina, L Peacock, M Raithatha, S Sarai, E Vilar, R Yellon.

Homerton University Hospital NHS Foundation Trust K Woods (PI), A Claxton (Co-PI), Y Akinfenwa, N Aladangady, H Bouattia, R Brady, R Corser, H Furreed, C Holbrook, S Jain, J Kaur, C Mitchell-Inwang, R Mullett, T Tanqueray, E Timlick,

Betsi Cadwaladr LHB: Ysbyty Gwynedd C Subbe (PI), N Boyle, C Butterworth, M Joishy, G Rieck, A Thomas.

Taunton and Somerset NHS Foundation Trust J Pepperell (PI), J Ashcroft, C Branfield, S Crouch, C Lanaghan, D Lewis, C Lorimer, H Mills, G Modgrill, A Moss, M Nixon, S Northover, K O'Brien, K Roberts, J Rogers, C Thompson, N Thorne, R Wallbutton, E Zebracki.

Guy's and St Thomas' NHS Foundation Trust H Winslow (PI), L Brace, K Brooks, L Chappell, M Flanagan, J Kenny, G Nishku, C Singh, E Wayman, C Williamson, H Winslow, C Yearwood Martin.

East Sussex Healthcare NHS Trust A Marshall (PI), S Blankley, H Brooke-Ball, T Christopherson, M Clark, T De Freitas, E De Sausmarez, D Hemsley, O Kankam, T Morley, A Newby, S Panthakalam, R Reddy, N Roberts, J Sinclair, R Venn, F Willson, TT Win, M Yakubi, A Zubir.

Betsi Cadwaladr LHB: Wrexham Maelor Hospital D Southern (PI), M Garton (Co-I), S Ahmer, G Bennett, S David, S Davies, E Heselden, M Howells, R Hughes, S Kelly, A Lloyd, H Maraj, H Reddy, S Robertson, G Spencer, G Szabo, S Tomlins.

Barnsley Hospital NHS Foundation Trust K Inweregbu (PI), M Cunningham, A Daniels, L Harrison, A Hassan, S Hope, M Hussain, A Khalil, S Meghjee, A Nicholson.

West Hertfordshire Hospitals NHS Trust V Page (PI), R Vancheeswaran (Co-PI), L Norris, T Varghese, X Zhao.

NHS Borders: Borders General Hospital A Scott (PI), S Alcorn, J Aldridge, J Bain, A Campbell, J Dawson, C Evans, C Flanders, N Hafiz, L Knox, J Lonnen, C Murton, B Muthukrishnan, F Rodger, B Soleimani, M Tolson.

Airedale NHS Foundation Trust T Gregory (PI), M Babirecki, H Bates, E Docks, E Dooks, F Farquhar, B Hairsine, S Nallapeta, S Packham.

NHS Lothian: St John's Hospital S Lynch (PI), S Begg, M Colmar, C Cheyne, R Frake, A Gatenby, C Geddie, F Guarino, C Kuronen-Stewart, A MacRaild, M Mancuso-Marcello, M Odam, OK Otite, L Primrose, A Saunderson, A Williams.

NHS Dumfries and Galloway: Dumfries & Galloway Royal Infirmary D Williams (PI), M McMahon (Co-PI), P Cannon, J Duignan, C Jardine, A Mitra, P Neill, S Wisdom.

NHS Ayrshire and Arran: University Hospital Ayr K Walker (PI), R Cuthbertson, J Locke, L McNeil, S Meehan, A Murphy, K Prasad, M Rodger, C Turley, S Walton.

Yeovil District Hospital NHS Foundation Trust A Broadley (PI), S Board, A Daxter, I Doig, A Getachew, L Howard, A Kubisz-Pudelko, A Lewis, K Mansi, B Mulhearn, A Shah, R Smith, D Wood.

Salford Royal NHS Foundation Trust P Dark (PI), C Bethan, B Blackledge, N Diar Bakerly, K Knowles, S Lee, T Marsden, J Perez, M Poulaka, R Sukla, M Taylor, V Thomas.

Belfast HSC Trust D Downey (PI), A Blythe, S Carr, D Comer, D Dawson, R Ingham, J Kidney, J Leggett, A Redfern-Walsh.

NHS Ayrshire and Arran: University Hospital Crosshouse A Clark (PI), T Adams, S Allen, K Bain, A Bal, C Burns, D Callaghan, N Connell, V Dey, F Elliott, K Gibson, D Gilmour, H Hartung, M Henry, G Houston, L McNeil, A Murphy, S Smith, S Walton, D Wilkin, M Wilson, S Wood.

Northern Devon Healthcare NHS Trust R Manhas (PI), U Akudo, A Attiq, V Ayra, C Baldwick, F Bellis, H Black, L Brunton, M Bryce, K Causer, S Cockburn, R Crowder, D Davies, C Ferreira-De Almeida, M Freeborn, H Goss, E Gray, I Gurung, G Hands, R Hartley, B Holbrook, N Hollister, R Horn, J Hunt, MS Jeelani, S Kyle, M Lamparski, M Lewis, S Ley, L Lindenbaum, S Mole, A Moody, J Morrison, J Raza, T Reynolds, G Rousseau, B Rowlands, M Ruiz, G Sacher, C Smith, D Tharmaratnam, B Theron, A Umeh, L van Koutrik, N Vernon, C White, E Willis.

NHS Highland B Sage (PI), F Barrett, W Beadles, A Cochrane, R Cooper, A Goh, S Makin, J Matheson, D McDonald, C Millar, K Monaghan, L Murray, D Patience, G Simpson.

Isle Of Wight NHS Trust M Pugh (PI), A Brown, S Grevatt, E Jenkins, S Knight, E Nicol, J Wilkins.

Torbay and South Devon NHS Foundation Trust T Clarke (PI), I Akinpelu, S Atkins, J Blackler, J Clouston, G Curnow, A Foulds, C Grondin, S Howlett, C Huggins, L Kyle, S Martin, W O'Rourke, A Redome, J Redome, J Turvey.

Harrogate and District NHS Foundation Trust A Kant (PI), C Taylor (Co-PI), A Amin, A Daly, SJ Foxton, E Lau, C Morgan, M Tripouki, L Wills.

South Warwickshire NHS Foundation Trust S Tso (PI), P Parsons (Co-PI), S Bird, C Bannon, R Browne, B Campbell, S Dhariwal, G Kakoullis, F Mackie, C O'Brien, K Webb.

Northern HSC Trust P Minnis (PI), J Burns, L Davidson, A Fryatt, J Gallagher, C McGoldrick, M McMaster.

Hywel Dda LHB: Prince Philip Hospital S Ghosh (PI), S Coetzee, K Davies, L O'Brien, Z Omar, CV Williams.

NHS Lanarkshire: University Hospital Hairmyres M Patel (PI), F Burton (Co-PI), D Bell, R Boyle, D Cairney, K Douglas, L Glass, E Lee, L Lennon, B Welsh.

The Royal Marsden NHS Foundation Trust K Tatham (PI), S Jhanji (Co-I), P Angelini, E Bancroft, E Black, A Dela Rosa, E Durie, M Hogben, I Leslie, A Okines, S Shepherd, N Taylor, S Wong.

The Hillingdon Hospitals NHS Foundation Trust S Kon (PI), T Bate, L Camrasa, A Danga, S Dubrey, J Ganapathi, B Haselden, M Holden, S-J Lam, G Landers, P Law, N Mahabir, N Malhan, M Nasseri, T Nishiyama, P Palanivelu, J Potter, S Ramraj, T Sugai, A Trivedi, D Wahab.

East Cheshire NHS Trust T Nagarajan (PI), M Holland, L Huhn, MA Husain, N Keenan, X Lee, L Wilkinson, K Wolffsohn.

Salisbury NHS Foundation Trust M Sinha (PI), A Anthony, L Bell, S Diment, S Gray, A Hawkins, M Johns, I Leadbitter, W Matimba-Mupaya, A Rand, S Salisbury, F Trim.

Royal Brompton & Harefield NHS Foundation Trust A Shah (PI), A Reed (Co-PI), A Aramburo, R Mordi, C Prendergast, P Rogers, N Soussi, J Wallen.

Western HSC Trust M Kelly (PI), D Concannon, D McClintock, V Mortland, N Smyth.

NHS Greater Glasgow and Clyde: Inverclyde Royal Hospital M Azharuddin (PI), H Papaconstantinou (Co-PI), D Cartwright, T McClay, E Murray, O Olukoya.

The Christie NHS Foundation Trust V Kasipandian (PI), A Binns, J King, P Mahjoob-Afag, R Mary-Genetu, P Nicola, A Patel, R Shotton, D Sutinyte.

Great Ormond Street Hospital For Children NHS Foundation Trust M Peters (PI), A Bamford, L Grandjean (Co-PI), E Abaleke, O Akinkugbe, H Belfield, G Jones, T McHugh, L O'Neill, S Ray, AL Tomas.

Hywel Dda LHB: Bronglais General Hospital M Hobrok (PI), D Asandei, R Loosley, D McKeogh, L Raisova, A Snell, H Tench, T Wareham, R Wolf-Roberts.

The Walton Centre NHS Foundation Trust R Davies (PI), H Arndt, E Hetherington.

Hywel Dda LHB: Withybush Hospital J Green (PI), R Hughes, C Macphee, H Thomas.

Alder Hey Children's NHS Foundation Trust D Hawcutt (PI), D Afolabi, K Allison, S McWilliam, L O'Malley, L Rad, N Rogers, P Sanderson, G Seddon, J Whitbread.

Birmingham Women's and Children's NHS Foundation Trust K Morris (PI), J Groves, K Hong, D Jyothish, S Sultan.

Velindre NHS Trust J Powell (PI), R Adams (Co-PI), A Jackson.

NHS Western Isles G Stanczuk (PI), I Garcia Deniz, S Klaczek, M Murdoch.

Sheffield Children's NHS Foundation Trust P Avram (PI), C Kerrison (sub PI), A Bellini, F Blakemore, S Borg, K Bourne, J Bryant, C Chambers, H Chisem, J Clemens, H Cook, P Dimitri, M Dockery, M Elfadil, S Gormley, D Hawley, A Howlett, A-M McMahon, J Nolan, B O'Shea, N Roe, J Sowter.

NHS Golden Jubilee National Hospital B Shelley (PI), V Irvine, F Thompson.

Liverpool Women's NHS Foundation Trust R McFarland (PI), P Corlett, C Cunningham, S Holt, J McKenzie, C Morgan, M Turner.

Dragon's Heart Hospital J Coulson (PI), B Moore.

Supplementary Methods

Study organization

The RECOVERY trial is an investigator-initiated, individually randomized, open-label, controlled trial to evaluate the efficacy and safety of a range of putative treatments in patients hospitalized with COVID-19. The trial was conducted at 176 National Health Service (NHS) hospital organizations in the United Kingdom. The trial was coordinated by a team drawn from the Clinical Trial Service Unit and the National Perinatal Epidemiology Clinical Trials Unit within the Nuffield Department of Population Health at University of Oxford, the trial sponsor. Support for local site activities was provided by the National Institute for Health Research Clinical Research Network.

Treatment supply to local sites was supported by National Health Service (NHS) England and Public Health England. Access to relevant routine health care and registry data was supported by NHS DigiTrials, the Intensive Care National Audit and Research Centre, Public Health Scotland, National Records Service of Scotland, and the Secure Anonymised Information Linkage (SAIL) at University of Swansea.

Protocol changes

RECOVERY is a randomized trial among patients hospitalized for COVID-19. All eligible patients receive usual standard of care in the participating hospital and are randomly allocated between no additional treatment and one of several active treatment arms. Over time, additional treatment arms have been added (see Table). In version 4.0 of the protocol, a second randomization was introduced for those trial participants with hypoxia (oxygen saturation <92% on air or receiving oxygen) and inflammation (C-reactive protein ≥75 mg/dL), comparing the addition of tocilizumab vs. control on top of the treatment assigned in the first randomization. In version 6.0, a factorial design was introduced to the first randomization such that participants were also randomized to convalescent plasma vs. no additional treatment. As outlined in the protocol, if one or more of the active treatments was not available at the hospital or is believed, by the attending clinician, to be contraindicated (or definitely indicated) for the specific patient, then random allocation was between the remaining treatment arms.

The original and final protocol are included in the supplementary material to this publication, together with summaries of the changes made.

Table. Protocol changes to treatment comparisons

Protocol version	Date	Randomization	Treatment arms
1.0	13-Mar-2020	Main (part A)	No additional treatment<br>Lopinavir-ritonavir<br>Low-dose corticosteroid<br>Nebulised Interferon-ß-1a (never activated)
2.0	23-Mar-2020	Main (part A)	No additional treatment<br>Lopinavir-ritonavir<br>Low-dose corticosteroid<br>Hydroxychloroquine
3.0	07-Apr-2020	Main (part A)	No additional treatment<br>Lopinavir-ritonavir<br>Low-dose corticosteroid<br>Hydroxychloroquine<br>Azithromycin

Protocol version	Date	Randomization	Treatment arms
4.0	14-Apr-2020	Main (part A)<br><br><br><br><br>Second[a]	No additional treatment<br>Lopinavir-ritonavir<br>Low-dose corticosteroid<br>Hydroxychloroquine<br>Azithromycin<br><br>No additional treatment<br>Tocilizumab
5.0	24-Apr-2020	-	(no change – extension to children <18 years old)
6.0	14-May-2020	Main (part A)<br><br><br><br><br>Main (part B factorial)<br><br>Second[a]	No additional treatment<br>Lopinavir-ritonavir<br>Low-dose corticosteroid[b]<br>Hydroxychloroquine[c]<br>Azithromycin<br><br>No additional treatment<br>Convalescent plasma<br><br>No additional treatment<br>Tocilizumab
7.0	18-Jun-2020	Main (part A)<br><br><br><br>Main (part B factorial)<br><br>Second[a]	No additional treatment<br>Lopinavir-ritonavir<br>Low-dose corticosteroid[b]<br>Azithromycin<br><br>No additional treatment<br>Convalescent plasma<br><br>No additional treatment<br>Tocilizumab

[a] for patients with (a) oxygen saturation <92% on air or requiring oxygen or children with significant systemic disease with persistent pyrexia; and (b) C-reactive protein ≥75 md/dL)
[b] enrolment of adults ceased 8 June 2020 as more than 2,000 patients had been recruited to the active arm
[c] enrolment ceased 5 June 2020 when the Data Monitoring Committee advised that the Chief Investigators review the unblinded data.

Supplementary statistical methods

Sample size

As stated in the protocol, appropriate sample sizes could not be estimated when the trial was being planned at the start of the COVID-19 pandemic. As the trial progressed, the Trial Steering Committee, blinded to the results of the study treatment comparisons, formed the view that if 28-day mortality was 20% then a comparison of at least 2000 patients allocated to active drug and 4000 to usual care alone would yield at least 90% power at two-sided P=0·01 to detect a proportional reduction of one-fifth (a clinically relevant absolute difference of 4 percentage points between the two arms).

Baseline-predicted risk

Baseline−predicted risk of 28-day mortality was estimated through the formula 100 x exp(a)/(1 + exp(a)), where a = −1·23 − 2·85 (if age <50) − 2·03 (if age 50−59) − 1·21 (if age 60−69) − 0·51 (if age 70−79) + 0·42 (if male) − 0·34 (if >7 days since symptom onset) + 0·86 (if on oxygen only at randomization) + 2·18 (if on invasive mechanical ventilation at randomization) − 0·01 (if history of diabetes) + 0·22 (if history of heart disease) + 0·21 (if history of chronic lung disease) + 0·50 (if history of kidney disease). These regression coefficients were derived from a multivariable logistic regression model using data from all trial participants who (at the time of data-lock) had complete 28-day mortality follow-up data. The regression model additionally adjusted for treatment allocation (with usual care designated the reference category) and for all possible two-way interactions between the above baseline characteristics and treatment allocation. These additional terms were ignored when calculating baseline-predicted risk, however, in order to ensure that the estimates corresponded to risk *if assigned usual care*. Patients were then subdivided into three approximately equally-sized groups (across all RECOVERY participants) on the basis of their predicted risk: <30%, ≥30% to <45%, and ≥45%. It should be noted that the *sole* purpose of the model was to discriminate risk among patients in the trial (which it does very well, as can be seen by the differences in mortality rates seen across the three risk groups in Figure 3) so that the effects of treatment among patients at different levels of risk could be evaluated. It is not meant to be externally-valid and should not be used to predict mortality risk in future patients (in the UK or elsewhere).

Ascertainment and classification of study outcomes

Information on baseline characteristics and study outcomes was collected through a combination of electronic case report forms (see below) completed by members of the local research team at each participating hospital and linkage to National Health Service, clinical audit, and other relevant health records. Full details are provided in the RECOVERY Definition and Derivation of Baseline Characteristics and Outcomes Document which was published online (www.recoverytrial.net) on 9 June 2020.

Randomisation form

The Randomisation form (shown below) was completed by trained study staff. It collected baseline information about the participant (including demographics, COVID-19 history, comorbidities and suitability for the study treatments) and availability of the study treatments. Once completed and electronically signed, the treatment allocation was displayed.

The following modifications were made to the Randomisation form during the trial:

Randomisation form version	Date of release	Major modifications from previous version
1.0	19-Mar-20	Initial version (protocol V1.0)
2.0	25-Mar-20	For protocol V2.0<br>• Hydroxycholoroquine added as treatment<br>• Known long QT syndrome added to comorbidities<br>• Severe depression removed from comorbidities
3.0	09-Apr-20	For protocol V3.0<br>• Azithromycin added as treatment<br>• Suspected SARS-CoV-2 infection included in eligibility criteria
[Second randomisation form introduced]	23-Apr-20	For protocol 4.0<br>• Eligibility criteria for second randomisation<br>• Tocilizumab vs control as treatment allocations
4.0	09-May-20	For protocol V5.0<br>• Age ≥18 years removed from eligibility criteria<br>• Additional questions on child's age and weight added
5.0	21-May-20	For protocol V6.0<br>• Convalescent plasma added as treatment
6.0	28-May-20	Baseline use of remdesivir
7.0	01-Jul-20	For protocol V7.0<br>• Participants eligible if convalescent plasma is only available and suitable treatment

RECOVERY
Randomised Evaluation of COVID-19 Therapy

Test version only (v6.08 - 05/06/20)

Randomisation Program

Call Freefone **0800 138 5451** to contact the RECOVERY team for **URGENT** problems using the Randomisation Program or for medical advice.
All **NON-URGENT queries** should be emailed to **recoverytrial@ndph.ox.ac.uk**

Logged in as: **Barts Health NHS Trust**

Section A: Baseline and Eligibility

Date and time of randomisation: 5 Jun 2020 13:32

Treating clinician

A1. Name of treating clinician

Patient details

A2. Patient surname

Patient forename

A3. NHS number ☐ Tick if not available

A4. What is the patient's date of birth? / /

A5. What is the patient's sex?

Inclusion criteria

A6. Has consent been taken in line with the protocol?
If answer is No patient cannot be enrolled in the study

A7. Does the patient have proven or suspected SARS-CoV-2 infection?
If answer is No patient cannot be enrolled in the study

A8. Does the patient have any medical history that might, in the opinion of the attending clinician, put the patient at significant risk if they were to participate in the trial?

A8B. Is the patient willing to receive convalescent plasma?

A9. COVID-19 symptom onset date: / /

A10. Date of hospitalisation: / /

A11. Does the patient require oxygen?

A12. Does the patient **CURRENTLY** require ventilation or ECMO?
Invasive mechanical ventilation or extra-corporeal membrane oxygenation

Does the patient have any CURRENT comorbidities or other medical problems?

A13.1 Diabetes

A13.2 Heart disease

A13.3 Chronic lung disease

A13.4 Tuberculosis

A13.5 HIV

A13.6 Severe liver disease

A13.7 Severe kidney impairment (eGFR<30 or on dialysis)

A13.8 Known long QT syndrome

A13.9 Current treatment with macrolide antibiotics which are to continue
Macrolide antibiotics include clarithromycin, azithromycin and erythromycin

A13.10 Previous adverse reaction to blood or blood product transfusion

Are the following treatments UNSUITABLE for the patient?
If you answer Yes it means you think this participant should NOT receive this drug.

A14.1 Lopinavir-Ritonavir

A14.3 Azithromycin

A14B.1 Convalescent plasma

Are the following treatments available?

A15.1 Lopinavir-Ritonavir

A15.3 Azithromycin

A15B.1 Convalescent plasma

Current medication

A16 Is the patient currently prescribed remdesivir?

Please sign off this form once complete

Surname:

Forename:

Professional email:

Continue

Cancel

Home

Follow-up form

The Follow-up form (shown on the next page) collected information on study treatment adherence (including both the randomised allocation and use of other study treatments), vital status (including date and provisional cause of death if available), hospitalisation status (including date of discharge), respiratory support received during the hospitalisation, occurrence of any major cardiac arrhythmias and renal replacement therapy received.

The following modifications were made to the Follow-up form during the trial:

**Follow-up form version**	**Date of release**	**Modifications from previous version**
1.0	30-Mar-20	Initial version
2.0	09-Apr-20	Information on other treatments used during admission:<br>• Azithromycin, IL-6 receptor antagonist<br>Fact and result of SARS-CoV-2 PCR test
3.0	09-Apr-20	Update to functionality; no changes to questions
4.0	23-Apr-20	Duration of treatments added
5.0	12-May-20	Capture of major cardiac arrhythmias added
6.0	28-May-20	Updates to wording of questions.<br>Information on other treatments used during admission:<br>• Remdesivir, convalescent plasma

Follow-up

Date of randomisation

Patient's date of birth

yyyy-mm-dd

1. Which of following treatment(s) did the patient definitely receive as part of their hospital admission after randomisation? *

(NB Include RECOVERY study-allocated drug, only if given, PLUS any of the other treatments if given as standard hospital care)

- ☐ No additional treatment
- ☐ Lopinavir-ritonavir
- ☐ Corticosteroid (dexamethasone, prednisolone or hydrocortisone)
- ☐ Hydroxychloroquine
- ☐ Azithromycin or other macrolide (eg, clarithromycin, erythromycin)
- ☐ Tocilizumab or sarilumab
- ☐ Remdesivir

The following questions only appear if the treatments have been allocated at randomisation

Please select number of days the patient received lopinavir-ritonavir

○ 1 ○ 2 ○ 3 ○ 4 ○ 5 ○ 6 ○ 7 ○ 8 ○ 9 ○ 10

Please select number of days the patient received corticosteroid (dexamethasone, prednisolone or hydrocortisone)

○ 1 ○ 2 ○ 3 ○ 4 ○ 5 ○ 6 ○ 7 ○ 8 ○ 9 ○ 10

Please select number of days the patient received hydroxychloroquine

○ 1 ○ 2 ○ 3 ○ 4 ○ 5 ○ 6 ○ 7 ○ 8 ○ 9 ○ 10

Please select number of days the patient received azithromycin

This question and the following question cannot both be zero

○ 0 ○ 1 ○ 2 ○ 3 ○ 4 ○ 5 ○ 6 ○ 7 ○ 8 ○ 9 ○ 10

Please select number of days the patient received other macrolides (eg, clarithromycin, erythromycin)

○ 0 ○ 1 ○ 2 ○ 3 ○ 4 ○ 5 ○ 6 ○ 7 ○ 8 ○ 9 ○ 10

Please select number of doses of tocilizumab or sarilumab the patient received

○ 1 ○ >1

Please select number of days the patient received remdesivir

1 2 3 4 5 6 7 8 9 10

» Convalescent Plasma

How many convalescent plasma infusions did the patient receive?

This is plasma given as part of trial, not any standard fresh frozen plasma or other blood products that the patient may have been given

0 1 2

Were any infusions stopped early for any reason ie, the patient did not receive the full amount?

Yes No

How many were stopped early?

1 2

» Health Status

2. Was a COVID-19 test done for this patient?

(If multiple tests were done, and the results were positive and negative, please tick Yes – positive result and Yes – negative result)

- Yes – positive result
- Yes – negative result
- Not done

3. What is the patient's vital status? *

- Alive
- Dead

3.1 What is the patient's current hospitalisation status?

Q3.1 is only completed if the patients is alive at Q3

- Inpatient
- Discharged

The patient has been enrolled in the trial for **NaN** days

3.1.1 Date follow-up form completed **Q3.1.1 is only completed if patient is still an inpatient at Q3**

yyyy-mm-dd

3.1.1 What was the date of discharge? Q3.1.1 is only completed if patient has been discharged at Q3

yyyy-mm-dd

*

3.1 What was the date of death? Q3.1.1 is only completed if patient has died at Q3

yyyy-mm-dd

3.2 What was the underlying cause of death? *

This can be obtained from the last entry in part 1 of the death certificate

- ◯ COVID-19
- ◯ Other infection
- ◯ Cardiovascular
- ◯ Other

Please give details

4. Did the patient require any form of assisted ventilation (ie, more than just supplementary oxygen)? *

- ◯ Yes
- ◯ No

Please answer the following questions:

4.1 For how many days did the patient require assisted ventilation? *

4.2 What type of ventilation did the patient receive?

	Yes	No	Unknown
**CPAP alone**	◯	◯	◯
**Non-invasive ventilation (eg, BiPAP)**	◯	◯	◯
**High-flow nasal oxygen (eg, AIRVO)**	◯	◯	◯
**Mechanical ventilation (intubation/tracheostomy)**	◯	◯	◯

ECMO	◯	◯	◯

Total number of days the patient received invasive mechanical ventilation (intubation/tracheostomy) (from randomisation until discharge/death/28 days after randomisation)

Complete if invasive mechanical ventilation (intubation/tracheostomy) is Yes

5. Has the participant been documented to have a NEW cardiac arrhythmia at any point since the main randomisation?

◯ Yes

◯ No

◯ Unknown

5.1 Please select all of the following which apply

If Q5 is answered Yes, you must select at least one option here

☐ Atrial flutter or atrial fibrillation

☐ Supraventricular tachycardia

☐ Ventricular tachycardia (including torsades de pointes)

☐ Ventricular fibrillation

☐ Atrioventricular block requiring intervention (eg, cardiac pacing)

6. Did the patient require use of renal dialysis or haemofiltration?

◯ Yes

◯ No

7. Please enter UKOSS case ID if known *

Enter the full UKOSS case ID ie, COR_123

Complete only if patient was pregnant at randomisation

(select if you do not know the UKOSS case ID)

☐ Not known

Interim analyses: role of the Data Monitoring Committee

The independent Data Monitoring Committee reviews unblinded analyses of the study data and any other information considered relevant at intervals of around 2 weeks. The committee is charged with determining if, in their view, the randomized comparisons in the study provide evidence on mortality that is strong enough (with a range of uncertainty around the results that was narrow enough) to affect national and global treatment strategies. In such a circumstance, the Committee would inform the Steering Committee who would make the results available to the public and amend the trial arms accordingly. Unless that happened, the Steering Committee, investigators, and all others involved in the trial would remain blind to the interim results until 28 days after the last patient had been randomized to a particular intervention arm. Further details about the role and membership of the independent Data Monitoring Committee are provided in the protocol.

The Data Monitoring Committee determined that to consider recommending stopping a treatment early for benefit would require at least a 3 to 3·5 standard error reduction in mortality. The Committee concluded that examinations of the data at every 10% (or even 5%) of the total data would lead to only a marginal increase in the overall type I error rate.

Supplementary Tables

Webtable 1: Baseline characteristics of patients considered unsuitable for randomisation to lopinavir-ritonavir compared with those randomised to lopinavir-ritonavir versus usual care

	Randomised (n=5040)	Unsuitable (n=3063)
Age, years	66.2 (15.9)	67.4 (15.3)
<70	2830 (56%)	1649 (54%)
≥70 to <80	1027 (20%)	694 (23%)
≥80	1183 (23%)	720 (24%)
Sex		
Male	3077 (61%)	1947 (64%)
Female	1963 (39%)	1116 (36%)
Ethnicity		
White	3781 (75%)	2279 (74%)
Black, Asian, and Minority Ethnic	865 (17%)	486 (16%)
Unknown	394 (8%)	298 (10%)
Number of days since symptom onset	8 (4-12)	8 (4-14)
Number of days since hospitalisation	2 (1-4)	3 (1-6)
Respiratory support received		
No oxygen received	1321 (26%)	811 (26%)
Oxygen only	3515 (70%)	1525 (50%)
Invasive mechanical ventilation	204 (4%)	727 (24%)
Previous diseases		
Diabetes	1388 (28%)	910 (30%)
Heart disease	1311 (26%)	1050 (34%)
Chronic lung disease	1162 (23%)	750 (24%)
Tuberculosis	16 (<1%)	12 (<1%)
HIV	6 (<1%)	49 (2%)
Severe liver disease	0 (0%)	185 (6%)
Severe kidney impairment	376 (7%)	357 (12%)
Any of the above	2880 (57%)	2049 (67%)
SARS-Cov-2 test result		
Positive	4423 (88%)	2697 (88%)
Negative	595 (12%)	353 (12%)
Unknown	22 (<1%)	13 (<1%)

Results are count (%), mean (SD), or median (inter-quartile range). The 'oxygen only' group includes non-invasive ventilation. Severe liver disease defined as requiring ongoing specialist care. Severe kidney impairment defined as estimated glomerular filtration rate <30 mL/min/1.73m².

Webtable 2: Treatments given, by randomized allocation

	Treatment allocation	
	**Lopinavir-ritonavir (n=1616)**	**Usual care (n=3424)**
Compliance data available	1603	3410
Lopinavir-ritonavir received	1394 (87%)	5 (<1%)
Other treatments received		
Dexamethasone	160 (10%)	355 (10%)
Hydroxychloroquine	5 (<1%)	10 (<1%)
Azithromycin or other macrolide	374 (23%)	862 (25%)
Tocilizumab or sarilumab	41 (3%)	123 (4%)
Remdesivir	6 (<1%)	22 (<1%)
Not recorded	3 (<1%)	2 (<1%)

Percentages are of those with a completed follow-up form. Of those allocated lopinavir-ritonavir who received at least one dose, 61% received all (or nearly all) of their scheduled doses during their hospital stay (missing at most 1 day of treatment) while 77% received at least half of their scheduled doses.

Webtable 3: Effect of allocation to lopinavir-ritonavir on cause-specific 28-day mortality

	Treatment allocation	
**Cause of death**	**Lopinavir-ritonavir (n=1616)**	**Usual care (n=3424)**
COVID	326 (20.2%)	704 (20.6%)
Other infection	6 (0.4%)	7 (0.2%)
Cardiac	6 (0.4%)	4 (0.1%)
Stroke	1 (0.1%)	3 (0.1%)
Other vascular	2 (0.1%)	3 (0.1%)
Cancer	9 (0.6%)	19 (0.6%)
Other medical	23 (1.4%)	26 (0.8%)
External	1 (0.1%)	0 (0.0%)
Unknown cause	0 (0.0%)	1 (0.0%)
**Total: 28-day mortality**	**374 (23.1%)**	**767 (22.4%)**

Webtable 4: Effect of allocation to lopinavir-ritonavir on cardiac arrhythmia

	Treatment allocation	
	**Lopinavir-ritonavir (n=1616)**	**Usual care (n=3424)**
Number with follow-up form*	877	1771
Atrial flutter or atrial fibrillation	27 (3.1%)	60 (3.4%)
Other supraventricular tachycardia	6 (0.7%)	17 (1.0%)
Subtotal: Supraventricular tachycardia	33 (3.8%)	73 (4.1%)
Ventricular tachycardia	1 (0.1%)	8 (0.5%)
Ventricular fibrillation	2 (0.2%)	1 (0.1%)
Subtotal: Ventricular tachycardia or fibrillation	2 (0.2%)	9 (0.5%)
Atrioventricular block requiring intervention	1 (0.1%)	2 (0.1%)
**Total: Any major cardiac arrhythmia**	**36 (4.1%)**	**82 (4.6%)**

* Information on new cardiac arrhythmias was only collected on follow-up forms from 12 May 2020 onwards; percentages are of those with such a form completed.
